# Pantethine and Neurodegeneration: A Coenzyme A-Centered Framework Linking Metabolism, Neuroinflammation, and Mitochondrial Dysfunction

**DOI:** 10.3390/ijms27219926

**Published:** 2026-11-05

**Authors:** Otávio Augusto Garcia Simili, Gabriel Magno de Carvalho, Raissa Santana Dias Simões, Claudia Rucco Penteado Detregiachi, Eliana de Souza Bastos Mazuqueli Pereira, Virgínia Maria Cavallari Strozze Catharin, Giovani Augusto da Silva, Vitor B. Miola, Anupam Bishayee, Vitor E. Valenti, László Vécsei, Masaru Tanaka, Sandra Maria Barbalho

**Affiliations:** 1 Department of Biochemistry and Pharmacology, School of Medicine, University of Marília (UNIMAR), Marília 17525-902, SP, Brazil; otavio.simili@unimar.br (O.A.G.S.); gabriel.magno@unimar.br (G.M.d.C.); raissa.simoes@unimar.br (R.S.D.S.); claudia.detregiachi@unimar.br (C.R.P.D.); eliana.bastos@unimar.br (E.d.S.B.M.P.); virginia.catharim@unimar.br (V.M.C.S.C.); 2 Postgraduate Program in Structural and Functional Interactions in Rehabilitation, University of Marília (UNIMAR), Marília 17525-902, SP, Brazil; giovani.silva@unimar.br (G.A.d.S.); vitor.miola@unimar.br (V.B.M.); 3 Department of Pharmacology, College of Osteopathic Medicine, Lake Erie College of Osteopathic Medicine, Bradenton, FL 34211, USA; abishayee@lecom.edu; 4 Systematic Reviews and Meta-Analyses Center, School of Philosophy and Sciences, São Paulo State University, Marília 17525-900, SP, Brazil; vitor.valenti@unesp.br; 5 Department of Neurology, Albert Szent-Györgyi Medical School, University of Szeged, H-6725 Szeged, Hungary; vecsei.laszlo@med.u-szeged.hu; 6 HUN-REN-SZTE Neuroscience Research Group, Danube Neuroscience Research Laboratory, Hungarian Research Network, University of Szeged (HUN-REN-SZTE), H-6725 Szeged, Hungary; 7 Research Coordination, UNIMAR Charity Hospital, School of Medicine, University of Marília (UNIMAR), Marília 17525-902, SP, Brazil

**Keywords:** pantethine, coenzyme A, kynurenine, neurodegeneration, mitochondrial dysfunction, neuroinflammation, oxidative stress, glial cells, pantothenate kinase-associated neurodegeneration (PKAN), Alzheimer’s disease, Parkinson’s disease

## Abstract

Neurodegenerative diseases are a growing global health burden associated with aging and characterized by progressive neuronal dysfunction, metabolic failure, mitochondrial impairment, oxidative stress, and chronic neuroinflammation. Among the metabolic pathways implicated in these disorders, coenzyme A (CoA)-linked biology has emerged as a potentially important but still underexplored contributor to neuronal resilience and vulnerability. Pantethine, a disulfide derivative of pantetheine and a CoA-related metabolic precursor, has attracted attention because of its reported effects on cellular metabolism, redox balance, and inflammatory signaling. However, its relevance across neurodegenerative diseases remains unevenly defined, with direct support strongest in pantothenate kinase-associated neurodegeneration (PKAN) and more limited evidence in common disorders such as Alzheimer’s disease (AD) and Parkinson’s disease (PD). This narrative review critically examines the mechanistic and translational evidence linking pantethine to neurodegeneration. PKAN represents the most logical disease context for pantethine investigation because impaired CoA biosynthesis is proximal to disease pathogenesis, although pantethine remains investigational and its clinical efficacy has not been established. By contrast, proposed applications in AD and PD remain highly theoretical and hypothesis-generating. Nevertheless, research on pantethine and related CoA-restoring strategies may identify new intervention targets across neurodegenerative diseases and other disorders characterized by impaired cellular bioenergetics, including selected neuropsychiatric disorders. These possibilities require biomarker-informed, disease-specific studies that establish active-species exposure, target engagement, and clinically meaningful effects.

## 1. Introduction

Aging is a progressive and multifactorial biological process marked by the gradual accumulation of molecular and cellular alterations, including genomic instability, telomere attrition, epigenetic remodeling, mitochondrial dysfunction, impaired proteostasis, and stem cell exhaustion [1,2]. These hallmarks are associated with increased vulnerability to chronic non-communicable diseases, particularly those affecting metabolic and neurological systems [3]. Demographic projections further intensify this concern, with the proportion of the global population aged 65 years or older expected to rise substantially by 2050 [4]. As longevity increases, preserving quality of life becomes a major biomedical and public health priority. This issue is especially pressing in disorders that progressively erode cognition, movement, autonomy, and social functioning [5].

Neurodegenerative diseases are therefore emerging as one of the defining medical challenges of aging societies [6]. They comprise a heterogeneous group of chronic and progressive conditions characterized by selective neuronal vulnerability, synaptic dysfunction, and gradual loss of neuronal structure and function [7]. Among them, Parkinson’s disease (PD) and Alzheimer’s disease (AD) are the most prevalent in older adults [8]. PD is primarily associated with degeneration of dopaminergic neurons in the substantia nigra and with the accumulation of alpha-synuclein (α-synuclein)-containing Lewy bodies [9,10]. AD, the leading cause of dementia, is characterized by progressive cognitive decline accompanied by amyloid-beta (amyloid-β) deposition, tau pathology, glial dysregulation, and broad metabolic disturbances. Although these disorders differ in clinical presentation and proteinopathic signatures, they share chronic neuroinflammatory signaling and microglial dysregulation [11,12,13,14], together with broader abnormalities in bioenergetics, mitochondrial function, oxidative stress, and cellular metabolism [9,15,16].

**Figure 1:**
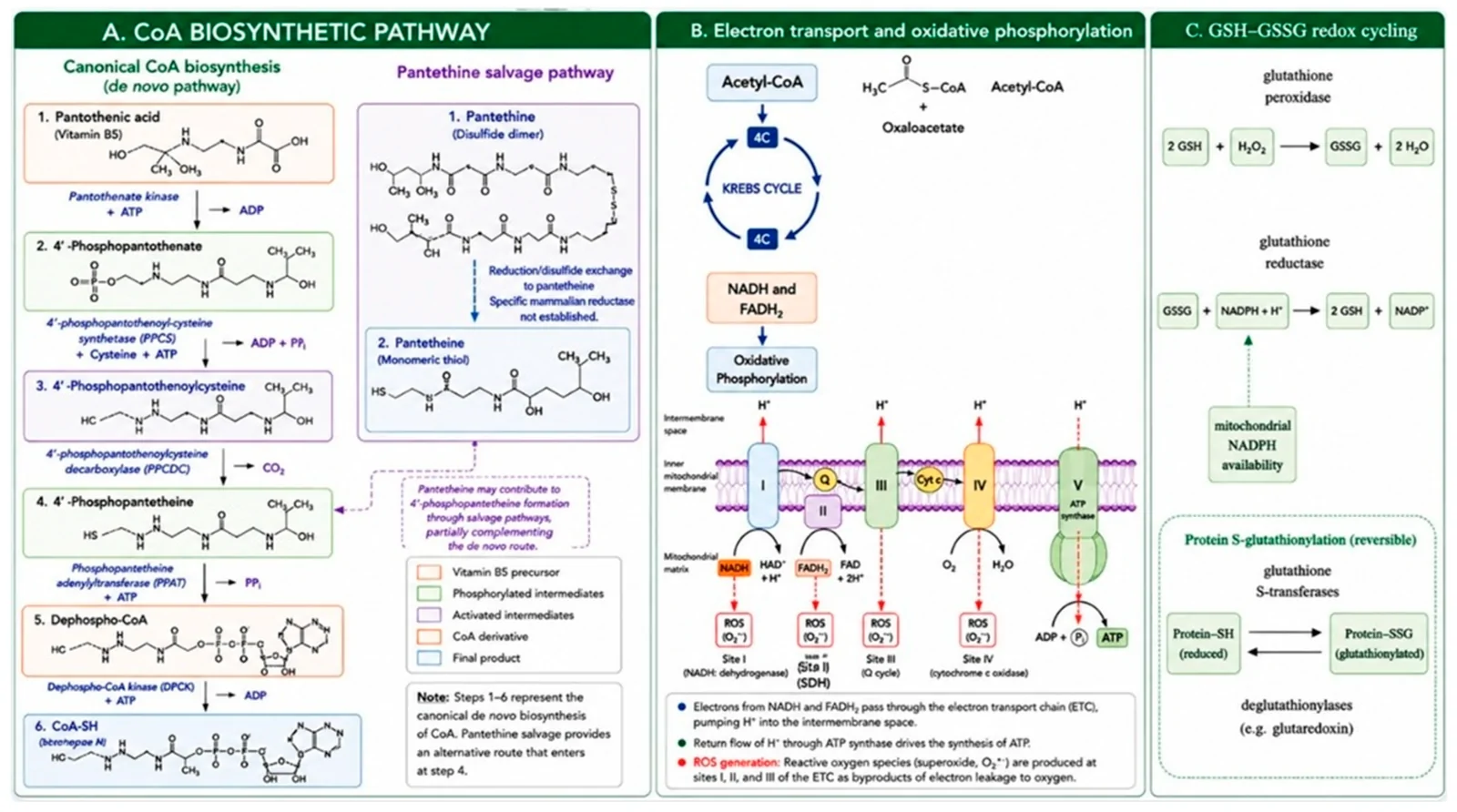
Position of pantethine within CoA-related metabolism. Canonical CoA biosynthesis proceeds from pantothenate through PANK-dependent phosphorylation and subsequent enzymatic steps to CoA. Pantethine can undergo reduction or disulfide exchange to pantetheine; however, a specific mammalian pantethine reductase responsible for this conversion has not been conclusively established, and the extent to which pantetheine contributes to downstream CoA salvage is context-dependent. This relationship should not be interpreted as equivalent to direct bypass of the PANK2-catalyzed step by 4′-phosphopantetheine. Downstream consequences of CoA availability for acetyl-CoA production, mitochondrial metabolism, and redox homeostasis are indicated schematically rather than by reproducing the complete TCA cycle, electron-transport chain, or glutathione redox network. ATP, adenosine triphosphate; ADP, adenosine diphosphate; AMP, adenosine monophosphate; CoA, coenzyme A; CoA-SH, reduced coenzyme A; CO_2_, carbon dioxide; DPCK, dephospho-CoA kinase; ETC, electron transport chain; FADH_2_, reduced flavin adenine dinucleotide; GSH, reduced glutathione; GSSG, oxidized glutathione; H_2_O_2_, hydrogen peroxide; NADH, reduced nicotinamide adenine dinucleotide; NAD^+^, oxidized nicotinamide adenine dinucleotide; NADPH, reduced nicotinamide adenine dinucleotide phosphate; NADP^+^, oxidized nicotinamide adenine dinucleotide phosphate; PP_i_, inorganic pyrophosphate; PPCS, 4′-phosphopantothenoylcysteine synthetase; PPCDC, 4′-phosphopantothenoylcysteine decarboxylase; PPAT, phosphopantetheine adenylyltransferase; ROS, reactive oxygen species; TCA, tricarboxylic acid.

Coenzyme A (CoA) is central to several of these processes. It is required for central carbon metabolism, fatty acid oxidation and synthesis, acetylation reactions, neurotransmitter-related metabolism, and mitochondrial energy production [17]. In neurons, disturbances in CoA-dependent pathways may have broad consequences because energetic demand is high and tolerance for metabolic instability is limited [18]. Reduced CoA availability may impair acetyl-CoA generation, tricarboxylic acid (TCA)-cycle activity, lipid handling, membrane homeostasis, and redox balance [19]. In turn, these changes may contribute to synaptic dysfunction, neurotransmitter imbalance, and progressive neuronal injury [20]. CoA-linked metabolism provides a mechanistic connection among bioenergetic failure, inflammatory stress, and mitochondrial dysfunction [21] and may contribute to neurodegenerative pathogenesis [22,23,24]. Pantethine is biochemically linked to this framework through its relationship to CoA and acetyl-CoA metabolism, connecting substrate utilization with mitochondrial bioenergetics and cellular redox homeostasis [22,25] (Figure 1).

Interest in CoA biology is especially strong in pantothenate kinase (PANK)-associated neurodegeneration (PKAN), a rare inherited disorder caused by mutations in PANK2, a key enzyme in CoA biosynthesis [26]. PKAN provides one of the clearest examples of how disruption of CoA homeostasis can be linked to neurodegenerative pathology, including mitochondrial abnormalities, iron dyshomeostasis, and profound metabolic dysfunction [27,28]. Because PKAN is mechanistically tied to the CoA biosynthetic pathway itself, it offers the most direct disease framework in which CoA-related interventions can be examined [29]. At the same time, broader age-related neurodegenerative disorders such as AD and PD are not defined by primary defects in CoA synthesis [30]. This distinction limits direct extrapolation from PKAN to AD and PD. It means that evidence arising from PKAN may be highly informative mechanistically yet cannot be transferred automatically to more common neurodegenerative diseases without careful qualification [27,31,32].

Pantethine is a pantothenic acid-derived compound closely linked to CoA biosynthesis [23,33]. Through CoA-related metabolism, it has been associated with acetyl-CoA production, fatty acid metabolism, membrane lipid turnover, and redox regulation [18,19,20]. These interconnected metabolic effects provide the rationale for investigating pantethine in disorders characterized by metabolic instability. Its appeal lies less in a single mechanism than in the possibility that it may influence several interconnected processes that are repeatedly implicated in neurodegeneration [22,23,34,35], although it is not an established neuroprotective therapy.

Experimental evidence indicates that pantethine can influence mitochondrial, redox, and inflammatory pathways, but the strength of support varies substantially by disease context [21,22,23,29]. Mechanistic relevance is strongest in PKAN and related inherited CoA-biosynthesis disorders, whereas evidence in AD and PD remains predominantly preclinical and hypothesis-generating [36,37,38]. Accordingly, this review distinguishes disease-proximal findings from broader mechanistic inference and evaluates translational claims according to model relevance, pharmacology, and the availability of human evidence [39,40,41].

This review is organized around a single question: how strongly does current evidence support pantethine as a CoA-centered modifier of neurodegenerative biology? The discussion therefore proceeds from pantethine pharmacology and CoA homeostasis to disease-proximal evidence in PKAN, followed by the more exploratory evidence relevant to AD and PD. Neuroinflammation, redox biology, mitochondrial dysfunction, and cell-survival pathways are considered where they provide mechanistic context for this central framework. Findings from related compounds or non-neural systems are distinguished from pantethine-specific evidence. This structure is intended to separate established biochemical relationships from disease-relevant inference and translational hypotheses.

## 2. From Mechanism to Translation: An Evidence-Based Appraisal

The evidence is evaluated along two distinct dimensions. First, mechanistic or disease proximity describes how directly a model connects the intervention to the disease-defining biological defect; findings are therefore categorized as disease-proximal, disease-relevant, mechanistically indirect, or hypothesis-generating. Second, evidentiary characteristics are considered independently according to experimental system, intervention specificity, CNS relevance, human relevance, and major methodological limitations (Supplementary Table S1). These dimensions should not be conflated. For example, a Drosophila dPANK/fumble model is disease-proximal because it directly models PANK deficiency, while its non-mammalian nature limits clinical generalizability. Conversely, 5xFAD astrocytes provide mammalian, disease-relevant pantethine evidence but do not model a primary CoA-biosynthesis defect. Table 1 summarizes these distinctions across the principal mechanistic domains and disease contexts.

### 2.1. Pantethine Pharmacology and CoA-Related Metabolism

#### 2.1.1. Pantethine as a CoA-Related Metabolic Compound

Pantethine is the stable disulfide form of pantetheine, an intermediate linked to CoA metabolism and salvage [57,58]. Although pantethine is metabolically related to downstream CoA intermediates, this relationship should not be interpreted as evidence that pantethine itself bypasses the PANK2-catalyzed step. Its contribution to CoA restoration is likely to depend on tissue-specific processing, pathway context, and residual biosynthetic capacity [22,23,35] (Figure 1A).

CoA is indispensable for acyl-group transfer, fatty acid oxidation and synthesis, acetyl-CoA formation, membrane lipid metabolism, and broader bioenergetic regulation [25]. These processes are tightly integrated. As a result, changes in CoA availability can affect mitochondrial respiration, lipid remodeling, and redox balance at the same time [59]. This is especially relevant in tissues with high metabolic demand, where relatively modest shifts in acetyl-CoA and acyl-CoA pools may have wide biological consequences [60].

Beyond its mechanistic relevance, pantethine has a substantial clinical history as a lipid-modifying agent. In Japan, oral pantethine is approved for selected cases of hyperlipidemia, whereas clinical studies conducted in Europe, North America, and China have reported reductions in total cholesterol, low-density lipoprotein cholesterol, non-high-density lipoprotein cholesterol, apolipoprotein B, or triglycerides. The magnitude and pattern of response vary according to dose, dyslipidemia phenotype, dietary intervention, and study design. Regulatory status also differs internationally. Pantethine is generally marketed as a dietary supplement in the United States and as a natural health product in Canada, while pantethine-specific lipid-lowering medicines have not been confirmed through centralized European, United Kingdom, or Australian registers. Lipid-profile modification therefore represents its most established clinical application, although cardiovascular-event prevention has not been demonstrated [61,62,63,64,65,66].

Pantethine has also been investigated for broader effects on inflammatory tone, stress adaptation, and cellular metabolism [23]. Even so, its biologic significance cannot be inferred from pathway proximity alone. Interpretation therefore requires distinguishing pantethine itself from the metabolites generated during its processing and considering whether the relevant effects arise from CoA repletion, cysteamine formation, or broader systemic metabolic changes.

#### 2.1.2. Pantethine, Pantetheine, and Cysteamine: Metabolic Relationships and Active-Species Uncertainty

A central challenge in interpreting pantethine biology is that pantethine, pantetheine, and cysteamine are metabolically linked but functionally distinct. Pantethine is the disulfide form of pantetheine. Pantetheine, in turn, is the immediate substrate for pantetheinases of the vanin family, which hydrolyze it into pantothenate and cysteamine as part of the CoA salvage pathway [67]. This means that once pantethine is administered, the biologically relevant species may depend on where hydrolysis occurs, how rapidly it proceeds, and which compartments have access to the resulting intermediates [59]. The pathway is therefore branching rather than strictly linear.

This branching has direct pharmacologic consequences [59]. If pantethine is extensively cleaved before it reaches the tissue of interest, then observed biologic effects may reflect indirect replenishment of upstream precursors, cysteamine-mediated redox actions, altered lipid metabolism, or a combination of these mechanisms rather than direct intracellular delivery of pantethine itself [68]. Human data are particularly instructive. In cystinotic children, oral pantethine was rapidly cleaved by pantetheinase in the gut and plasma, circulating pantethine was undetectable, and the major measurable products were pantothenate and cysteamine. That result does not eliminate therapeutic relevance, but it strongly cautions against assuming that the administered molecule remains the dominant active species in vivo. Rat studies similarly showed that oral pantethine leads to substantial pantothenate recovery in blood and urine, again indicating efficient absorption coupled to partial hydrolysis during or after gastrointestinal transit [69]. Older human excretion data also support the view that pantethine is handled differently from pantothenic acid, but do not establish intact tissue exposure directly [70].

Brain-relevant evidence reinforces this uncertainty. In hippocampal slice cultures, extracellular metabolism studies showed that pantetheinase converts cystamine to cysteamine very efficiently, whereas conversion of pantethine is much more limited under the tested conditions [43]. Together, these findings indicate that extracellular cysteamine generation is substrate-dependent, while related rat studies suggest that the lower potency of pantethine may reflect limited brain pantetheinase activity [71,72]. Accordingly, pantethine should not be discussed as though its downstream products arise uniformly across tissues [59].

Vanin biology adds another layer of complexity. Vanin 1 (VNN1) and related pantetheinases sit at the crossroads of CoA recycling, redox regulation, and inflammatory signaling [73]. In some systems, vanin activity supports metabolic adaptation and mitochondrial oxidative function by linking pantetheine turnover to CoA and acetyl-CoA metabolism [59]. In others, cysteamine production influences oxidative stress responses, epithelial resilience, and inflammatory behavior [74]. Neuroendocrine studies suggest that pantethine, possibly through intracellular cysteamine formation, may reduce somatostatin and prolactin levels in the cerebral cortex and hypothalamus, although the mechanism remains uncertain [75]. In rats, pantethine dose-dependently suppressed open-field activity, altered hypothalamic noradrenaline, dopamine, and 3,4-dihydroxyphenylacetic acid levels, and attenuated somatostatin-induced barrel rotation; cysteamine was more potent at equimolar doses [76]. A separate time-course study showed that these behavioral, monoaminergic, and striatal somatostatin effects were transient and largely resolved within 24 h [77]. Pharmacologic inhibition studies also show that disrupting vanin activity alters fasting lipid profiles, underscoring the systemic metabolic relevance of this pathway [78]. It remains uncertain whether the observed biological effects reflect direct CNS delivery of pantethine, downstream metabolites such as pantetheine or cysteamine, peripheral immunometabolic modulation, or a combination of these mechanisms [59,79].

#### 2.1.3. CoA Homeostasis, Acetyl-CoA Biology, and Neurodegenerative Vulnerability

The rationale for considering pantethine in neurodegeneration rests on the centrality of CoA homeostasis [24]. CoA is not simply a housekeeping cofactor [27]. It is a determinant of metabolic flexibility, nutrient-state adaptation, acyl-group trafficking, and integration between mitochondrial and cytosolic metabolism [59]. Acetyl-CoA, one of its most important derivatives, is equally central [80]. It supports TCA flux, ATP production, lipid synthesis, and acetylation reactions that influence cellular structure and signaling [80]. In neurons and glia, where energetic demand is high and reserve capacity is limited, disturbances in CoA and acetyl-CoA biology can propagate across several systems at once [22,81,82].

This has direct relevance to neurodegenerative vulnerability [83]. Neural cells depend on tightly regulated mitochondrial function, membrane turnover, neurotransmitter-related metabolism, and stress adaptation, all of which intersect with CoA-dependent pathways [24]. Reduced CoA availability may limit acetyl-CoA generation, constrain TCA activity, impair lipid remodeling, and weaken redox buffering capacity [80] (Figure 1A,C). The broader CoA literature links disturbed CoA homeostasis to neurodegeneration, cardiometabolic dysfunction, myopathy, and maladaptive stress states, indicating that CoA insufficiency can be pathogenic rather than merely associative [23]. This view is strengthened by evidence that CoA metabolism is dynamically regulated rather than static [19]. Phosphoinositide 3-kinase (PI3K) and protein kinase B (AKT) signaling influence de novo CoA synthesis through PANK-related control, including phosphorylation-dependent regulation of pantothenate kinase 4 (PANK4), thereby altering acetyl-CoA and acyl-CoA pools and reshaping lipid metabolism [84]. In other words, CoA biology responds to nutrient and growth signaling and can sit upstream of broad metabolic phenotypes [81,85,86].

The strongest disease-specific rationale emerges in inherited CoA-biosynthesis disorders [27,29]. In PKAN, mutations in PANK2 impair an early step in CoA synthesis, making CoA pathway dysfunction proximal to pathogenesis rather than secondary to downstream degeneration [87]. It provides a biologically coherent context in which pantethine, pantothenate, or related intermediates might help improve downstream CoA availability, particularly when residual pathway activity persists [45]. In such settings, a CoA-centered intervention is not merely plausible. It is mechanistically anchored [27,36,45].

At the same time, the significance of CoA biology extends beyond rare Mendelian disease [27]. Experimental work in diverse tissues suggests that CoA availability influences mitochondrial function, lipid handling, inflammatory tone, and adaptation to metabolic stress [88]. CoA-linked dysfunction may contribute to broader metabolic failure in AD and PD, but this mechanistic association does not establish disease modification by pantethine [26,83]. Evidence that pantethine alters disease-relevant CoA biology in the appropriate CNS compartment and produces meaningful neural effects remains incomplete [87,88].

#### 2.1.4. Blood–Brain Barrier Permeability and Unresolved CNS Target Engagement

A major translational uncertainty in pantethine research is whether orally or systemically administered pantethine reaches the central nervous system (CNS) in its intact form, or whether most observed effects instead reflect peripheral metabolism and downstream products [42]. This pharmacokinetic uncertainty directly affects interpretation of CNS efficacy [89]. This challenge reflects a broader principle in CNS drug development: mechanistic promise cannot be translated reliably unless molecular properties, formulation, administration route, transporter interactions, and brain-exposure measurements are considered together [90]. It shapes how one interprets efficacy claims, model-specific findings, and the broader leap from PKAN to common neurodegenerative diseases [91].

Available data suggest that pantethine is metabolically labile [42]. Rat studies showed that oral pantethine increases circulating and urinary pantothenate, consistent with efficient gastrointestinal absorption coupled to substantial mucosal hydrolysis [69]. Human data point in the same direction [42]. In cystinosis, circulating pantethine was undetectable after oral dosing, whereas pantothenate and cysteamine rose markedly, indicating rapid extra-CNS conversion of the administered compound. Urinary studies in humans support differential handling of pantethine relative to pantothenic acid, but do not establish intact brain exposure [69].

Together, these findings suggest that oral pantethine may have limited opportunity to cross the BBB in substantial intact form unless it escapes rapid degradation [36]. Administered dose should therefore not be equated with intact brain exposure [50]. Because pantethine undergoes substantial extra-CNS metabolism, increasing the oral dose may increase systemic exposure to pantothenate, pantetheine-derived products, or cysteamine without producing a proportional increase in intact pantethine within the CNS. Dose–response studies should therefore quantify the parent compound and major metabolites in plasma and, where feasible, cerebrospinal fluid or brain tissue, and relate these exposure measures to CoA-pathway target engagement [92]. This distinction is essential because an absence of efficacy could reflect inadequate CNS exposure rather than failure of the underlying metabolic mechanism [56].

This uncertainty creates several possibilities [59]. Intact pantethine may reach the brain only minimally, with apparent CNS effects arising largely from peripheral metabolic changes or from downstream metabolites with different transport properties [93]. Alternatively, local salvage pathways involving pantetheine, pantothenate, or related intermediates may contribute to compartment-specific CoA repletion if suitable precursors become available [59]. Work in yeast shows that alternative precursor uptake routes can support CoA synthesis under constrained conditions [94]. Those findings cannot be mapped directly onto the mammalian brain, but they reinforce the broader principle that precursor form, transporter availability, and compartment-specific metabolism are all likely to matter [94].

**Figure 2:**
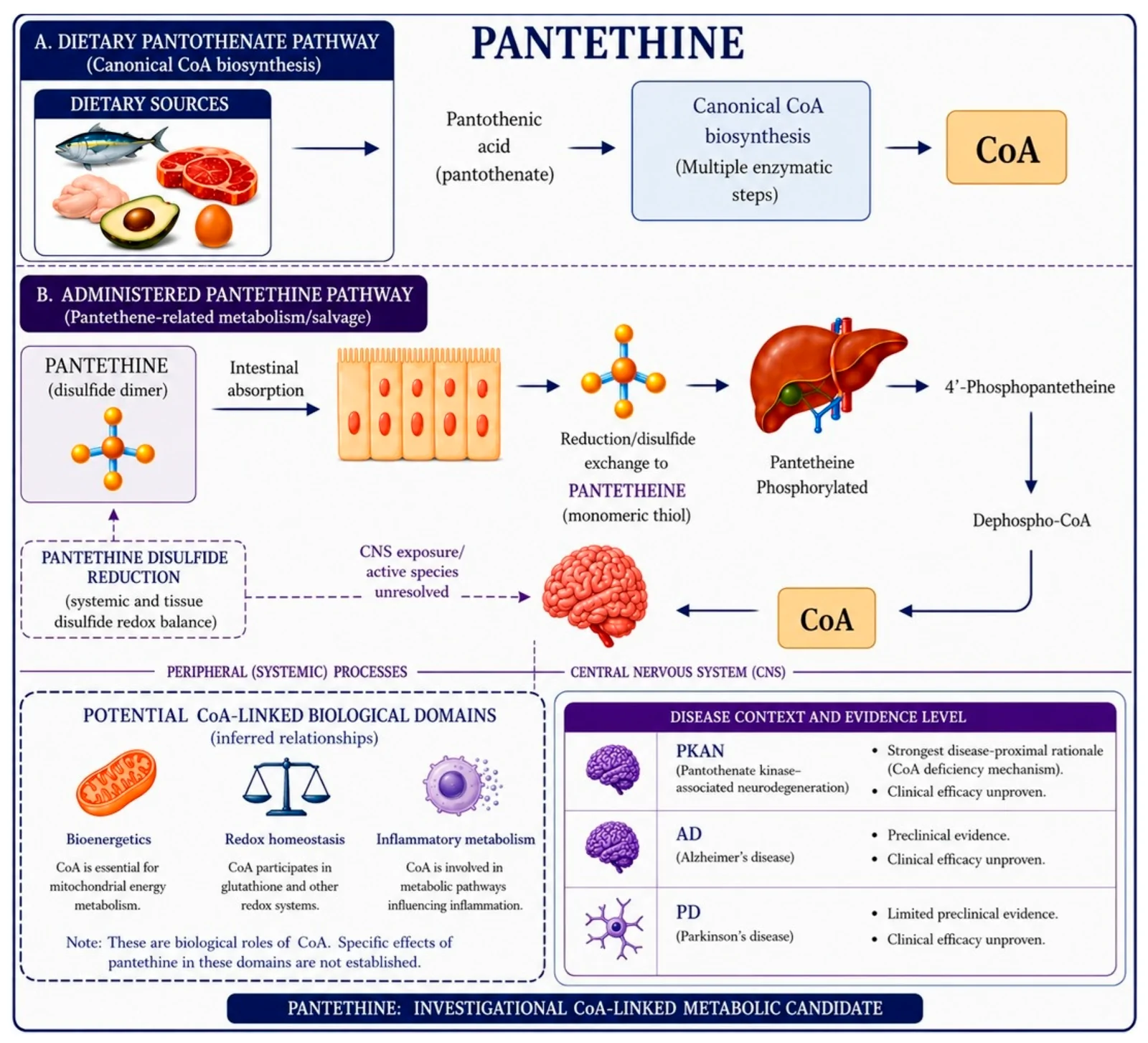
Dietary pantothenate is incorporated into canonical CoA biosynthesis through multiple enzymatic steps. In contrast, administered pantethine, a disulfide dimer, undergoes intestinal absorption and reduction/disulfide exchange to pantetheine, which can enter pantetheine-related metabolism and CoA salvage pathways, ultimately contributing to CoA availability. Pantethine or CoA-related mechanisms have been investigated in AD, PD, and PKAN. Pantethine is presented as an investigational CoA-linked metabolic candidate, rather than as an established therapeutic intervention. AD, Alzheimer’s disease; ATP, adenosine triphosphate; CoA, coenzyme A; PD, Parkinson’s disease.

Interpretation therefore differs by disease context [50]. In PKAN, indirect metabolic support may remain relevant because CoA-pathway dysfunction is disease-proximal, even if intact pantethine delivery to the brain is limited. Extrapolation to AD and PD is less direct because systemic metabolic effects cannot be assumed to produce CNS disease modification [36,95]. The extent to which pantethine restores brain CoA directly, acts through pantetheine or cysteamine, or produces mainly peripheral immunometabolic effects remains unresolved. Figure 2 summarizes these pharmacologic uncertainties and distinguishes PKAN, AD, and PD by evidentiary strength, while Table 1 provides the broader evidence hierarchy.

### 2.2. Evidence Hierarchy Across Neurodegenerative Contexts

#### 2.2.1. Direct Evidence from PKAN and Inherited CoA-Biosynthesis Disorders

The strongest evidence linking pantethine-related strategies to neurodegeneration comes from inherited CoA-biosynthesis disorders, particularly PKAN and disorders involving PANK2 or COASY dysfunction [27]. In these conditions, CoA-pathway impairment is mechanistically proximal to pathogenesis rather than a secondary disease correlate [87], allowing precursor-based or bypass-oriented interventions to be evaluated within a defined biological framework [27,31,32,96]. Extending this rationale beyond PKAN, fibroblasts and human induced pluripotent stem-cell-derived astrocytes from patients with COASY protein-associated neurodegeneration exhibited iron overload, mitochondrial abnormalities, impaired vesicular trafficking, lipid peroxidation, ferroptosis-related changes, and cellular senescence, with astrocytes more closely reproducing the disease-associated phenotype than fibroblasts [97]. Further support for pantethine-responsive CoA restoration comes from phosphopantothenoylcysteine synthetase deficiency. Pathogenic PPCS variants reduce cellular CoA and impair cardiomyocyte contractility, whereas pantethine partially rescues functional abnormalities in patient-derived cardiac models and has been associated with sustained clinical improvement. Because these findings primarily concern cardiomyopathy, they should be regarded as supportive evidence for correction of inherited CoA-pathway dysfunction rather than direct evidence of neuroprotection.

This logic is supported by convergent evidence across cellular, animal, and human-derived neuronal systems. PANK-deficient Drosophila models show reduced CoA, mitochondrial dysfunction, oxidative stress, neurodegeneration, and shortened lifespan, all of which are improved by pantethine feeding [36]. Consistent with these findings, pank2 knockdown in zebrafish severely disrupted anterior central nervous system and vascular development, whereas supplementation with pantethine or CoA, but not vitamin B5, efficiently rescued the developmental phenotype, underscoring the dependence of neural and vascular integrity on PANK2 activity and CoA homeostasis [98]. In mammalian PKAN models, 4′-phosphopantetheine corrects abnormalities in CoA, iron handling, dopamine metabolism, and mitochondrial enzyme activity in brain tissue and patient-derived cells [50]. Human induced pluripotent stem cell (iPSC)-derived PANK2 neurons likewise show premature death, increased reactive oxygen species (ROS), mitochondrial dysfunction, and excitability defects that can be corrected by CoA supplementation [99]. Additional work in PANK2-mutant cells suggests that pantethine-containing supplement regimens can partially normalize iron accumulation, mitochondrial markers, and oxidative phenotypes when residual PANK2 activity is present [38,45,100]. Even so, the PKAN literature also illustrates the importance of pharmacologic realism. Clinical translation has been uneven, and BBB penetration remains a decisive constraint for any CoA-pathway therapy. Still, among all disease contexts considered here, PKAN provides the most direct mechanistic rationale for pantethine-related discussion [27,36,101].

#### 2.2.2. Intermediate Mechanistic Evidence from Inflammatory and Metabolic Models

A second tier of evidence comes from models that do not represent primary CoA-biosynthesis disorders but nevertheless show that CoA-related metabolism can shape mitochondrial function, inflammatory signaling, iron handling, or oxidative balance in ways relevant to neurodegeneration [24]. This evidence is mechanistically informative but interpretively intermediate. It supports biological plausibility without establishing disease-specific therapeutic efficacy [27].

Such models are useful because they clarify how impaired CoA homeostasis can perturb cellular metabolism beyond rare Mendelian disease [27]. Reviews of brain CoA and acetyl-CoA metabolism, for example, emphasize that disturbances in CoA-dependent pathways can affect mitochondrial enzymes, glutathione stability, inflammatory tone, and neuronal resilience under stress [83]. Experimental data from Coasy-deficient neural lineages further support a causal relationship between CoA pathway disruption and neurodevelopmental as well as neurodegenerative phenotypes, including impaired mitochondrial respiration, lipid peroxidation, iron dysregulation, and chronic neuroinflammation [27]. These findings extend the mechanistic field of relevance, but they do not, by themselves, demonstrate that pantethine can reverse equivalent pathology in common neurodegenerative diseases. They are best interpreted as bridge evidence between direct PKAN biology and broader disease extrapolation [17,23,24].

#### 2.2.3. Exploratory Relevance to Alzheimer’s Disease and Parkinson’s Disease

Evidence for AD and PD is more indirect and remains largely preclinical [83]. A different evidentiary standard is therefore required [102] because pantethine is being considered against broader metabolic, inflammatory, and mitochondrial abnormalities rather than a primary defect in CoA biosynthesis [83].

In AD, this intersection is supported by several lines of evidence [103]. Brain studies have reported cerebral pantothenate deficiency together with reductions in CoA-dependent or CoA-modulated TCA enzymes, suggesting that impaired pantothenate and CoA metabolism may contribute to defective cerebral energetics. In parallel, pantethine has shown beneficial effects in preclinical AD models [37]. In 5xFAD astrocytes, it alleviates metabolic and inflammatory abnormalities, including IL-1β-related changes [46]. In transgenic mouse models, longer-term treatment reduces gliosis, amyloid burden, and behavioral deficits, while also reshaping inflammatory and synaptic gene expression profiles [37]. Additional data from triple-transgenic AD mice suggest improvement in cognition, inflammation, cholesterol handling, and gut microbiota composition. These findings are biologically coherent but remain preclinical [104,105,106,107].

The case for PD is even more tentative in the present evidence set [83]. This caution is consistent with the broader view of PD as a multifactorial systems disorder in which molecular, imaging, and clinical heterogeneity complicate biomarker standardization and therapeutic translation [102,108]. Reviews of brain CoA and acetyl-CoA biology describe pantothenate deficiency and CoA-related vulnerability in PD-relevant tissue, but direct pantethine evidence is far more limited than in PKAN or AD models [83]. Metabolomic evidence nevertheless suggests altered pantothenate status in PD, while experimental studies link pantethine-related ketone metabolism and TCA-cycle remodeling to dopaminergic vulnerability; these observations remain supportive rather than therapeutic evidence [24,109,110]. As a result, PD relevance remains largely hypothesis-generating and should not be placed on the same evidentiary footing as inherited CoA-biosynthesis disorders [109].

#### 2.2.4. Cross-Cutting Uncertainties and Limits of Translation

Across disease contexts, three uncertainties limit interpretation of pantethine-related findings: the identity of the biologically active molecular species, CNS exposure, and the scarcity of human efficacy data [38]. It remains unclear whether observed effects are mediated by pantethine itself, downstream metabolites, restoration of CoA homeostasis, or indirect systemic actions [37]. CNS delivery is also unresolved, particularly when peripheral or mixed-system findings are extrapolated to brain disease [111]. Finally, clinical evidence remains limited; even in PKAN, where the mechanistic rationale is strongest, preclinical rescue has not translated into unequivocal therapeutic efficacy [56]. Accordingly, support is strongest in PKAN and related inherited CoA-biosynthesis disorders, intermediate in disease-relevant metabolic and inflammatory models, and exploratory in AD and PD [110].

### 2.3. Pantethine and Neuroinflammation Signaling

#### 2.3.1. Neuroinflammation as a Convergent Feature of Neurodegeneration

Neuroinflammation is now recognized as a convergent and dynamic feature of many neurodegenerative disorders rather than a secondary by-product of neuronal loss alone [112]. In the CNS, microglia and astrocytes respond to aggregated proteins, altered metabolites, oxidative stress, and cell-derived danger signals by changing their transcriptional programs, cytokine output, and metabolic behavior [113]. In its early or regulated form, this response may support tissue surveillance, debris clearance, and transient repair [114]. When sustained, however, it can amplify synaptic dysfunction, BBB disturbance, oxidative injury, and neuronal vulnerability [115]. This dual role is especially relevant in disorders such as AD, PD, amyotrophic lateral sclerosis, and neurodegeneration with brain iron accumulation, where chronic inflammatory signaling often coexists with mitochondrial dysfunction and altered cellular metabolism [11,12,13]. In aging brains, persistent inflammatory signaling may also interact with declining neurogenesis and maladaptive microglial and astrocytic states, thereby weakening synaptic plasticity and cognitive resilience [116].

Chronic inflammatory signaling is closely linked to metabolic context [117]. Microglial and astrocytic phenotypes are shaped by nutrient availability, mitochondrial status, lipid handling, and redox balance [118]. Tryptophan and kynurenine metabolism further illustrate how peripheral metabolism, immune signaling, and brain function converge within inflammatory-metabolic pathways [119,120]. Pantethine should therefore be interpreted as a CoA-related metabolic precursor with possible effects on inflammatory state rather than as a conventional cytokine-targeting drug [121,122,123].

#### 2.3.2. Pantethine-Associated Effects on Inflammatory Mediators in Preclinical Systems

The most direct evidence linking pantethine to neuroinflammatory regulation comes from preclinical systems in which inflammatory and metabolic readouts were measured together [46]. In cultured astrocytes from the 5xFAD mouse model of AD, pantethine alleviated a pathological profile characterized by altered glycolysis and TCA activity, increased basal inflammatory tone, and marked elevation of IL-1β expression. Similar changes were induced by amyloid-β exposure in wild-type astrocytes, and pantethine significantly reduced both IL-1β mRNA and protein while also normalizing aspects of astrocyte activation and metabolism. These findings provide direct CNS-relevant evidence that pantethine can modify astrocytic inflammatory signaling under AD-like experimental conditions. They do not establish broad disease-modifying control of glial phenotypes across neurodegenerative disorders.

A second important line of evidence comes from experimental autoimmune neuroinflammation [34]. In encephalitogenic T cells from experimental autoimmune encephalomyelitis, CoA synthesis pathways differ from those in resting T cells, suggesting that inflammatory pathogenicity is coupled to altered CoA metabolism. Pantethine-mediated CoA fueling reduced proliferation, adhesion, and production of pro-inflammatory cytokines in myelin-specific T cells, while both prophylactic and therapeutic administration ameliorated disease severity in vivo. The same study also reported reduced pro-inflammatory cytokine production, including tumor necrosis factor alpha (TNF-α), GM-CSF, and interleukin 17A (IL-17A), in human Th1 and Th17 cells and in T cells from patients with multiple sclerosis. These findings are mechanistically significant because they show that pantethine can influence inflammatory outputs in both murine and human immune cells. At the same time, they should be interpreted primarily as evidence of immunometabolic modulation rather than as direct proof of microglial or astrocytic reprogramming in degenerating brain tissue.

The broader CoA-deficiency literature helps explain why such anti-inflammatory effects are biologically plausible [83]. Astrocyte-lineage-specific deletion of CoA synthase in mice leads to neurodevelopmental defects accompanied by impaired mitochondrial respiration, iron dyshomeostasis, lipid peroxidation, and chronic neuroinflammation [124]. Related reviews of inherited CoA-biosynthesis disorders describe reactive astrocytosis, activated microglia, and neuroinflammatory pathology in PKAN and COASY protein-associated neurodegeneration (CoPAN) brain regions, often alongside iron accumulation and mitochondrial dysfunction [27]. These observations do not demonstrate pantethine efficacy directly, but they strengthen the mechanistic rationale for testing CoA-related interventions in inflammatory neurodegenerative settings [26]. They suggest that chronic inflammation in these disorders may emerge not only from immune signaling itself, but also from persistent metabolic and mitochondrial disruption [24,27,97].

Pantethine has been associated with reduced inflammatory signaling and improved metabolic status in selected preclinical contexts, including AD-related astrocyte models and autoimmune neuroinflammation [34,37]. It may also indirectly influence glial inflammatory states through CoA-linked metabolism, redox balance, or mitochondrial performance [48]. Direct evidence for disease-specific suppression of microglial activation, normalization of astrocyte state transitions, or broad cytokine-network remodeling within the CNS remains limited [125]. Although pantethine has been linked to cytokine modulation in non-neural or mixed inflammatory settings, the brain-focused evidence for interleukin 8 (IL-8) is less direct than for IL-1β-related and T-cell-associated inflammatory outputs [126,127,128].

Similar caution applies to claims regarding an NADPH increase, glial quiescence, or reduced amyloid deposition [114]. Some downstream metabolic changes are plausible given the roles of CoA and acetyl-CoA in energy metabolism and redox support, but they should not be presented as established pantethine effects unless measured directly in disease-relevant CNS models [44]. In models showing reduced inflammatory pathology, the data are more consistent with modification of an inflammatory-metabolic state than with selective anti-cytokine activity [24].

#### 2.3.3. Limits of Current Evidence for Glial and Cytokine Modulation

Several limitations should therefore be made explicit [129]. First, the current literature does not justify treating pantethine as a broadly validated modulator of glial phenotypes across neurodegenerative diseases [130]. The strongest direct data come from cultured 5xFAD astrocytes and from immune-cell-centered models of experimental neuroinflammation [34]. These are important, but they do not capture the full spatial and temporal complexity of glial crosstalk in vivo [131,132]. Second, much of the rationale linking pantethine to neuroinflammation is still inferential and rests on the broader observation that CoA deficiency, mitochondrial dysfunction, iron dysregulation, and lipid peroxidation can sustain chronic inflammatory activation in the brain [124].

Third, cytokine claims should be weighed according to the model in which they were measured [133]. IL-1β reduction in astrocyte-based AD models and TNF-α, GM-CSF, and IL-17A reduction in encephalitogenic or human T cells are supported more directly than generalized claims about IL-8, broad microglial suppression, or disease-specific amyloid clearance by pantethine [34]. Whether these effects reflect pantethine itself, downstream metabolites, or broader CoA-related immunometabolic remodeling remains uncertain [134]. Pantethine therefore shows anti-inflammatory potential in selected preclinical contexts, but direct evidence for precise glial and cytokine reprogramming in neurodegenerative disease remains incomplete [34]. Similar evidence grading is required when interpreting oxidative, mitochondrial, and cell-survival effects [11,123].

**Figure 3:**
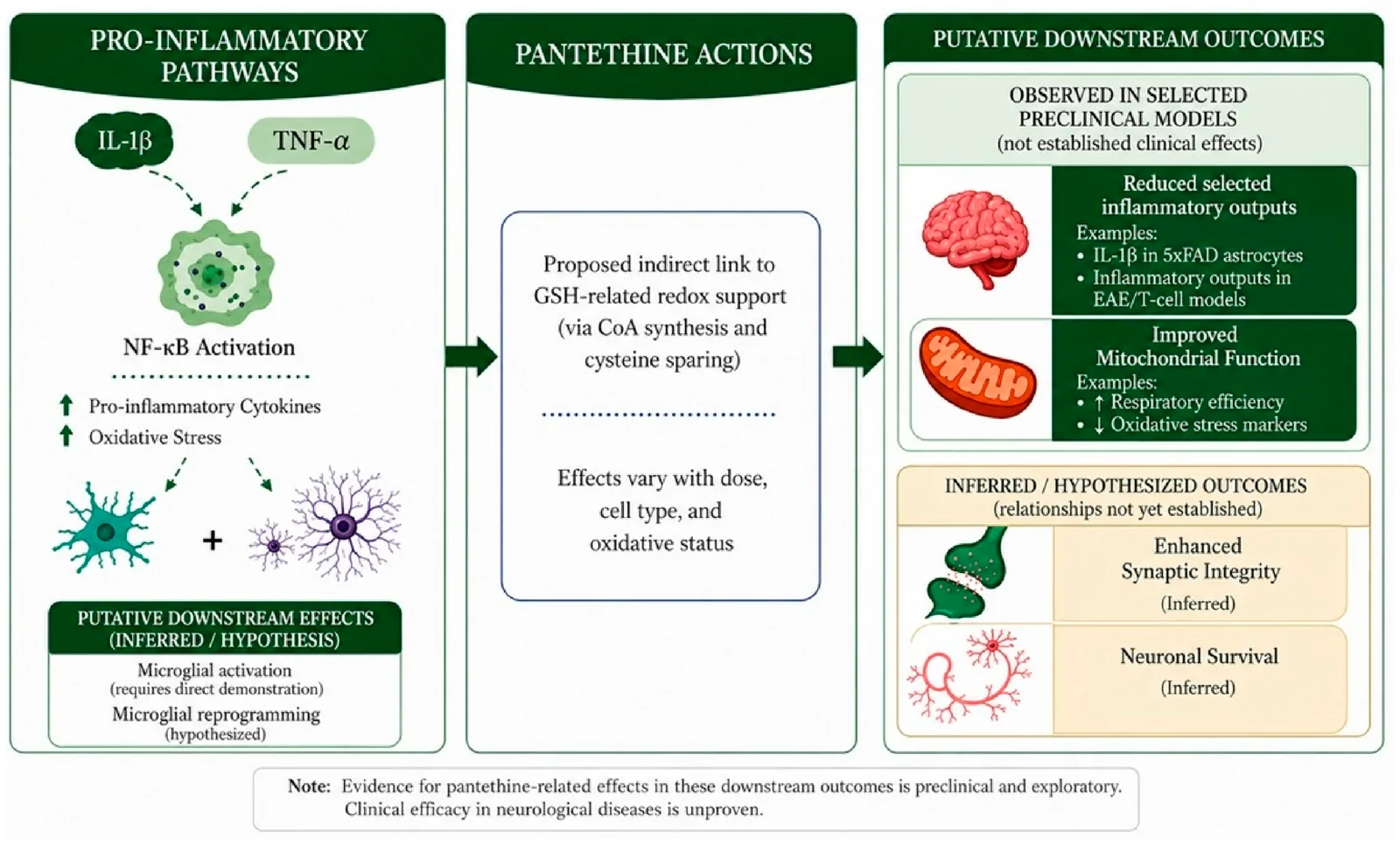
Proposed mechanistic framework for pantethine actions in the context of neuroinflammation. Pro-inflammatory stimuli, including interleukin-1β (IL-1β) and tumor necrosis factor-α (TNF-α), activate signaling pathways associated with inflammatory responses and oxidative stress, including nuclear factor kappa B (NF-κB)-related signaling. Pantethine has a proposed indirect link to glutathione (GSH)-related redox support through coenzyme A (CoA) synthesis and cysteine sparing; however, these relationships remain incompletely established and may vary according to dose, cell type, and oxidative status. In selected preclinical models, pantethine-related interventions have been associated with reduced selected inflammatory outputs, including IL-1β-related responses in 5xFAD astrocytes and inflammatory outputs in EAE/T-cell models, as well as improvements in selected indices of mitochondrial function and oxidative stress. By contrast, modulation of microglial activation or reprogramming, enhanced synaptic integrity, and neuronal survival should be considered inferred or hypothesis-generating downstream consequences unless directly demonstrated in the relevant experimental model. Overall, the framework distinguishes experimentally observed preclinical findings from proposed downstream effects. Clinical efficacy of pantethine in neurological diseases remains unproven. Abbreviations: CoA, coenzyme A; EAE, experimental autoimmune encephalomyelitis; GSH, reduced glutathione; IL-1β, interleukin-1β; NF-κB, nuclear factor kappa B; TNF-α, tumor necrosis factor alpha. ↑: Increase; ↓: Decrease.

These proposed relationships between inflammatory signaling, redox buffering, and downstream neuroprotective effects are summarized schematically in Figure 3.

### 2.4. Pantethine, Oxidative Stress, and Redox Homeostasis

#### 2.4.1. Redox Imbalance in Neurodegenerative Disease

In neurodegenerative disease, oxidative stress reflects disruption of redox homeostasis linked to mitochondrial dysfunction, impaired energy metabolism, and inflammatory signaling [135]. Excess reactive oxygen and nitrogen species can damage membrane lipids, proteins, and enzymes in neural tissue, where metabolic demand is high and regenerative reserve is limited [136]. Redox imbalance can also impair mitochondrial enzymes, destabilize glutathione-dependent defenses, alter protein thiol status, and amplify inflammatory injury [137,138,139,140].

Pantethine is relevant to this biology because of its links to CoA metabolism, thiol availability, glutathione homeostasis, and mitochondrial function [23,83]. Rather than acting primarily as a conventional radical scavenger, it may influence oxidative stress indirectly through thiol and mitochondrial pathways [83,141,142].

#### 2.4.2. Pantethine-Related Links to Glutathione and Antioxidant Defense

The strongest rationale for linking pantethine to redox homeostasis comes from its relationship to thiol metabolism and glutathione-dependent antioxidant defense [83]. Earlier work on pantothenic acid derivatives showed that pantothenic acid and pantothenol can protect mammalian cells from oxidative injury through mechanisms centered on glutathione and related intracellular thiol systems [143]. Although these studies do not establish pantethine-specific neuroprotection, they support the broader idea that CoA-related compounds can influence oxidative resilience through thiol maintenance rather than through simple direct scavenging [141,142].

More specific support comes from experimental systems in which pantethine or closely related derivatives were tested against oxidative disruption of brain tissue [143]. In isolated brain mitochondria exposed to oxidative stress, panthenol restored glutathione redox balance, reduced protein S-glutathionylation, improved mitochondrial energy metabolism, and inhibited lipid peroxidation [48,83]. When combined with succinate and N-acetylcysteine, these effects were even stronger, suggesting that the redox benefit depended not only on the precursor itself but also on the broader metabolic and glutathione-generating environment. Although these findings derive from panthenol rather than pantethine, they are relevant because they support the principle that CoA-related precursors can stabilize mitochondrial redox state in brain-relevant systems [138,144].

Evidence more directly involving pantethine comes from in vivo models of oxidative neurotoxicity [48]. In rotenone-treated rats, both panthenol and pantethine improved thiol-disulfide balance, restored glutathione redox status, reduced protein glutathionylation, and attenuated oxidative stress markers, particularly in basal ganglia structures vulnerable in Parkinsonian neurotoxicity. Similarly, in hippocampal oxidative injury induced by aluminum chloride, pantethine restored glutathione levels and redox potential, normalized glutathione-metabolizing enzymes, and corrected abnormal protein S-glutathionylation [49,145]. These studies are important because they connect pantethine-related treatment to brain tissue, mitochondrial stress, and glutathione-centered antioxidant defense rather than to peripheral redox systems alone.

These data support several nonexclusive mechanisms. Pantethine may improve redox buffering indirectly through CoA availability and mitochondrial metabolism [83] and may support glutathione-related antioxidant capacity through thiol-linked pathways [49]. Some apparent antioxidant effects may also result from improved energy status, reduced lipid peroxidation, and better enzyme function rather than direct chemical quenching of oxidants [145,146].

Additional support comes from PKAN-related systems, where oxidative stress is closely linked to defective CoA metabolism [51]. In PANK2-mutant cellular models, pantethine-containing supplement regimens reduced oxidative damage, lowered mitochondrial lipid peroxidation, and improved pyruvate dehydrogenase (PDH) and complex I activity [36,45]. In Drosophila models of PKAN, dietary pantethine restored CoA levels, improved mitochondrial function, reduced protein oxidation, and rescued neurodegenerative phenotypes [36]. Together, these studies suggest that pantethine-related redox benefits are especially credible in disease contexts where CoA insufficiency is mechanistically proximal [36,45,147].

By contrast, non-neural endothelial and fibrotic models should be interpreted more cautiously [148]. Pantethine reduced microparticle shedding and abolished oxidative and nitrosative stress in endothelial cells and fibroblasts, and related work implicated the pantethine-and-vanin-1 pathway in regulating ROS-driven fibrosis. These findings show that pantethine has biologically relevant redox effects outside the nervous system, but they do not by themselves establish neuronal or glial protection. Their main value here is supportive and mechanistic. They reinforce the view that pantethine can modify oxidative environments, while the neurodegenerative implications must still be inferred with restraint. The oxidative evidence summarized above is organized in Table 2, which distinguishes brain-relevant findings from supportive non-neural redox studies [148,149,150]; detailed concentrations, doses, routes, exposure durations, and other study-specific methodological information are provided in Supplementary Table S2.

Non-neural studies show pantethine-associated reductions in oxidative and nitrosative stress in endothelial and fibroblast systems, context-dependent cytoprotection in cancer-derived cells, and limited protection against cataract development in irradiated animals [148,151,153]. These findings remain supportive rather than direct evidence for neurodegeneration and are summarized in Table 2.

#### 2.4.3. Direct Evidence and Interpretive Limits in Neurodegeneration-Relevant Models

Pantethine and related CoA-linked precursors improve redox homeostasis in selected neurodegeneration-relevant models, particularly those involving glutathione imbalance, protein glutathionylation, mitochondrial dysfunction, and lipid peroxidation [48]. Evidence is strongest in brain tissue, PKAN-related models, and Parkinsonian toxin models, where pantethine is associated with restored glutathione redox balance, reduced oxidative damage, and improved mitochondrial enzyme activity [154].

However, not all supporting studies use pantethine itself [83]; some involve panthenol or other pantothenic acid derivatives. Antioxidant effects should not be equated with neuronal rescue unless neural outcomes were measured directly [155], and redox improvements may be secondary to improved metabolism rather than a distinct antioxidant mechanism [156]. Evidence from endothelial or fibrotic systems remains supportive rather than direct [151,157].

Overall, pantethine is best described as a metabolically linked redox modulator whose strongest effects occur in preclinical models coupling CoA deficiency, mitochondrial dysfunction, and glutathione imbalance [49,158].

### 2.5. Mitochondrial Dysfunction and Bioenergetic Rescue

#### 2.5.1. Mitochondrial Vulnerability in CoA-Linked Neurobiology

Mitochondria are especially vulnerable to CoA insufficiency because several core bioenergetic processes depend directly on CoA, acetyl-CoA, and phosphopantetheinylated mitochondrial proteins [83]. In neural tissue, this dependence is particularly consequential [19]. The brain relies on tightly regulated mitochondrial oxidative metabolism not only for adenosine triphosphate (ATP) production, but also for acetyl-CoA-dependent synthesis, redox buffering, and maintenance of specialized neuronal functions. For this reason, defects in CoA biosynthesis are expected to affect mitochondria early and disproportionately, rather than appearing only as downstream consequences of cell injury [82,87,140,159]. The biochemical position of pantethine relative to CoA metabolism and its principal downstream metabolic consequences are summarized in Figure 1.

PANK2 and COASY defects compromise mitochondrial CoA pools required for oxidative metabolism, lipoic acid-dependent enzyme activity, and mitochondrial acyl carrier protein (mtACP) function [19,51]. CoA deficiency is associated with reduced respiration, impaired PDH activity, altered membrane potential, defective iron-sulfur cluster biogenesis, and reduced ATP production [50]. These changes are direct consequences of disrupted CoA-linked mitochondrial metabolism and make mitochondrial dysfunction a major readout of pathway failure in PKAN and related disorders [24,27,147].

The strongest rescue data come from Pank2 models, Drosophila, fibroblasts, and other systems in which CoA-linked mitochondrial defects were measured directly [36]. Evidence for comparable effects in AD and PD remains limited.

#### 2.5.2. Direct Evidence for Mitochondrial Effects in PKAN-Related Models

The clearest evidence for mitochondrial rescue by pantethine or related CoA-restoring strategies comes from PKAN-linked models, where the disease mechanism itself is tied to impaired CoA biosynthesis [36]. This includes Drosophila systems, mammalian Pank2 models, human fibroblasts, and other cellular settings in which mitochondrial structure or function has been measured directly [147].

In dPANK- or fumble-deficient Drosophila, dietary pantethine restores CoA levels, improves mitochondrial function and morphology, rescues brain degeneration, enhances locomotion, and extends lifespan [36]. Because PANK deficiency directly impairs CoA synthesis, this model links pantethine rescue to a disease-proximal metabolic defect rather than to a nonspecific antioxidant effect. Related work links CoA deficiency to a CoA, mtACP, and PDH axis and suggests that pantethine-dependent salvage may be influenced by microbiome-mediated conversion to downstream intermediates [147]. These studies provide direct evidence that pantethine-related strategies can improve mitochondrial bioenergetics in a PKAN-relevant model [36].

Mammalian PKAN models support the same general conclusion, although with greater mechanistic granularity [147]. Pank2-null neurons and cells show reduced mitochondrial membrane potential, defective respiration, swollen or structurally altered mitochondria, and lower ATP content despite the absence of uniform loss across every respiratory complex [101,147]. These findings indicate a bioenergetic systems defect rather than a simple single-enzyme failure. Combined Pank1 and Pank2 deficiency in mouse brain similarly lowers CoA and short-chain acyl-CoAs, disrupts oxidative metabolism, and triggers transcriptional responses consistent with metabolic hypoxia [88]. This reinforces the idea that CoA depletion destabilizes mitochondrial metabolism at the level of integrated pathway flux [101,147].

Work with 4′-phosphopantetheine, which is not identical to pantethine but is relevant to the same CoA-restoration pathway, provides additional evidence [30]. In mammalian PKAN models, oral 4′-phosphopantetheine normalizes CoA, iron, dopamine biomarkers, and the activity of mitochondrial enzymes such as complex I and PDH [50]. Mechanistically, this appears to occur through restoration of mtACP function and related phosphopantetheinylated pathways. These findings demonstrate biochemical rescue of disease-linked mitochondrial readouts in brain tissue and patient-derived cells [99].

Importantly, 4′-phosphopantetheine and pantethine should not be regarded as pharmacologically equivalent CoA-restoring interventions. 4′-Phosphopantetheine enters CoA biosynthesis downstream of the PANK2-catalyzed phosphorylation step and therefore provides evidence that bypassing the PANK2 defect can restore downstream CoA-dependent functions. Pantethine does not establish the same bypass mechanism, and the available pantethine-containing supplementation data are most informative in settings in which residual PANK2 expression or activity persists. Consequently, rescue by 4′-phosphopantetheine should be interpreted as evidence for the tractability of downstream CoA restoration rather than as direct evidence that pantethine will be effective across all PANK2 genotypes. Mutation class and residual PANK2 activity may therefore be important determinants of pantethine responsiveness and should be incorporated into future experimental and clinical study design [47,53].

Human cell models also provide strong disease-proximal support [156]. PANK2-mutant fibroblasts show reduced mitochondrial phosphopantetheinyl-proteins, including mtACP, aminoadipate-semialdehyde synthase, and aldehyde dehydrogenase 1 family member L2, with downstream consequences for lipoic acid synthesis, complex I, PDH, and iron-sulfur cluster biology [160]. In responsive genotypes, supplementation strategies can recover these mitochondrial components and improve respiratory function [156]. Pantethine-containing supplement regimens in PKAN-mutant cells have also been reported to reduce iron accumulation, increase PANK2 and mtACP expression, restore complex I activity, and broadly improve mitochondrial pathology [160]. Related pilot work combining pantothenate, pantethine, omega-3 fatty acids, and vitamin E reported improved mtACP levels, PDH activity, and partial clinical stabilization in a small number of PKAN patients with residual enzyme function [29,38]. These findings should be interpreted cautiously because combination regimens do not isolate the specific contribution of pantethine [38]. Even so, they strengthen the case that CoA-centered rescue can improve mitochondrial readouts in selected PKAN settings [38,45,100].

Evidence from additional inherited CoA-biosynthesis disorders broadens this mitochondrial framework [161]. In a zebrafish model, pank2 knockdown disrupted nervous-system and vascular development, whereas pantethine or CoA, but not vitamin B5, efficiently rescued the developmental phenotype, supporting the importance of PANK2-dependent CoA homeostasis across tissues and species [45,98]. In phosphopantothenoylcysteine synthetase deficiency, pathogenic PPCS variants reduced cellular CoA and impaired mitochondrial and contractile function in patient-derived cardiac models, while pantethine partially restored cellular function and was associated with sustained clinical improvement [162]. Although the PPCS findings are not CNS-specific, they provide supportive human evidence that pantethine-responsive CoA restoration can improve organ dysfunction caused by an inherited CoA-biosynthesis defect [162]. To improve comparability across studies, Table S3 provides patient-specific pantethine dosing for the PPCS cohort, including the escalation of Patient 1’s dose from 450 to 900 mg/day, the highest fixed daily dose reported in this cohort.

Additional support comes from mtACP- and lipoic-acid-centered work [51]. Selective loss of mitochondrial phosphopantetheinyl proteins in PKAN fibroblasts tightly links CoA-pathway disruption to impaired respiration, while alpha-lipoic acid supplementation can increase PANK2, mtACP, lipoylated proteins, and restore PDH and complex I activities in responsive variants [163]. Although alpha-lipoic acid is not pantethine, these studies reinforce the central point that the mitochondrial phenotype in PKAN is mechanistically coherent and biochemically tractable. Pantethine should therefore be viewed within a broader family of CoA-restoring or CoA-bypassing strategies that converge on mitochondrial rescue [36]. Table 3 summarizes the mitochondrial evidence base across in vitro, in vivo, and disease-proximal experimental systems, highlighting where pantethine-related rescue is most direct and where interpretation remains supportive rather than definitive.

#### 2.5.3. Broader Mitochondrial Implications in Neurodegeneration: Plausible but Not Established

The broader relevance of these mitochondrial findings to common neurodegenerative diseases is biologically plausible but not yet established at the same level of confidence [27]. Reviews of brain CoA and acetyl-CoA metabolism emphasize that mitochondrial CoA pools are essential for oxidative metabolism, acetylcholine synthesis, N-acetylaspartate production, and redox protection, and that CoA precursors can stabilize mitochondrial function in toxin and neuroinflammation models [83]. Related reviews of PKAN, CoPAN, and other inborn errors of CoA metabolism likewise identify mitochondrial dysfunction and oxidative stress as recurring consequences of impaired CoA synthesis in barrier-protected, high-demand tissues such as the brain [87]. This broader literature makes it reasonable to hypothesize that mitochondrial support is one of the main ways pantethine-related interventions could influence neurodegenerative biology [24,82,158,159].

Among PD-relevant experimental systems, the MPTP/MPP^+^ model provides the most direct pantethine-specific mitochondrial evidence identified in this review. In C57BL/6 mice exposed to 2′-methyl-MPTP, pantethine preserved mitochondrial respiratory-chain complex I activity, maintained brain glutathione, supported mitochondrial ATP production, and restored ketone-related metabolic changes associated with dopaminergic injury. Pantethine treatment was also associated with protection against dopaminergic neuronal loss and motor impairment. These findings provide mechanistically relevant evidence that pantethine can modify mitochondrial and redox dysfunction in a PD-related toxin model. However, MPTP/MPP^+^ produces an acute toxin-induced mitochondrial lesion and does not reproduce the genetic, proteinopathic, and slowly progressive biology of idiopathic PD; the findings therefore support a PD-relevant mechanism rather than disease-modifying efficacy in patients [113].

At the same time, important boundaries must be maintained [27]. Most direct rescue data come from inherited CoA-biosynthesis disorders or experimental systems designed around CoA deficiency [50]. In these settings, mitochondrial dysfunction is mechanistically proximal, and CoA-restoring strategies address a defined metabolic bottleneck. In AD, PD, and other common neurodegenerative conditions, mitochondrial dysfunction is certainly relevant, but the causal chain is more complex and less specific to the CoA pathway [18]. Other metabolic pathways can also generate mitochondrial and redox vulnerability; for example, KAT II deletion produces regional kynurenine remodeling with oxidative and excitotoxic imbalance, while kynurenine-receptor signaling has been linked to ATP maintenance, calcium handling, mitophagy, redox defense, and inflammasome control [165,166]. One possible, although still indirect, link involves ketone-body metabolism, as pantethine has been reported to increase L-3-hydroxybutyryl-CoA dehydrogenase activity and circulating ketone-body levels in an experimental context relevant to dopaminergic injury, while ketone bodies can function as alternative mitochondrial fuels and metabolic signals under conditions of energetic stress [110,164]. As a result, evidence that pantethine improves mitochondrial function in PKAN models should not be presented as proof that equivalent rescue occurs in common neurodegenerative diseases in vivo [105,109,167]. Thus, pantethine-related mitochondrial rescue is most persuasive in PKAN-proximal systems and remains exploratory for common neurodegenerative diseases [50].

A further, indirect metabolic intersection concerns de novo NAD^+^ synthesis through the kynurenine pathway [168]. CoA and NAD^+^/NADH make complementary contributions to mitochondrial bioenergetics, including TCA-cycle flux and electron transport [169]. However, there is currently no evidence that pantethine directly regulates the principal kynurenine-pathway enzymes [59]. This connection should therefore be regarded as systems-level metabolic crosstalk rather than a specific mechanism of pantethine action [170].

The kynurenine (KYN) pathway is the major route of tryptophan (Trp) catabolism and generates both neuroactive metabolites and substrates for de novo nicotinamide adenine dinucleotide (NAD^+^) synthesis. Tryptophan 2,3-dioxygenase (TDO), indoleamine 2,3-dioxygenase 1 (IDO1) and indoleamine 2,3-dioxygenase 2 (IDO2) initiate the metabolic pathway by converting tryptophan (Trp) to N-formylkynurenine, which arylformamidase subsequently converts to KYN. KYN then forms a major metabolic branch point. Kynurenine aminotransferases I to IV generate kynurenic acid (KYNA), whereas kynurenine 3-monooxygenase directs KYN toward 3-hydroxykynurenine (3-HK). Kynureninase converts KYN to anthranilic acid (AA) or 3-HK to 3-hydroxyanthranilic acid (3-HAA). The latter is converted by 3-hydroxyanthranilate 3,4-dioxygenase to 2-amino-3-carboxymuconate semialdehyde, which may cyclize spontaneously to quinolinic acid (QA). By contrast, 2-amino-3-carboxymuconate semialdehyde decarboxylase diverts pathway flux away from QA formation [171,172,173,174].

Importantly, KYNA is not an intermediate in de novo NAD^+^ synthesis. The NAD^+^ branch proceeds through 3-HK, 3-hydroxyanthranilic acid, and QA. Quinolinate phosphoribosyltransferase converts QA to nicotinic acid mononucleotide, after which nicotinamide mononucleotide adenylyltransferases generate nicotinic acid adenine dinucleotide and NAD synthetase produces NAD^+^. The resulting NAD^+^/NADH couple supports electron-transfer reactions during glycolysis, pyruvate oxidation, and the TCA cycle, while NADH supplies reducing equivalents to mitochondrial complex I. CoA and NAD^+^ therefore make complementary rather than interchangeable contributions to bioenergetics. CoA supports acyl-group transfer and acetyl-CoA formation, whereas NAD^+^/NADH couples substrate oxidation to mitochondrial electron transport [168,175,176,177,178] (Figure 1A,B).

Pathway distribution may also influence neurodegenerative vulnerability. Inflammatory induction of IDOs or altered kynurenine 3-monooxygenase activity can shift metabolism toward 3-HK and QA, thereby linking immune activation with oxidative, excitotoxic, and mitochondrial stress. Conversely, kynurenine aminotransferase-dependent KYNA formation influences glutamatergic and other receptor-mediated signaling, which intersects with synaptic plasticity and intrinsic excitability, and may modify mitochondrial stress responses [166,179,180,181,182,183,184]. Nevertheless, the literature reviewed here does not demonstrate that pantethine directly regulates the principal KYN-pathway enzymes. The relationship should therefore be framed as metabolic crosstalk: pantethine-related support of CoA metabolism and KYN-pathway-derived NAD^+^ may converge on TCA-cycle activity, electron transport, and redox homeostasis without establishing a direct causal interaction.

### 2.6. Pantethine and Cell Survival Pathways

#### 2.6.1. Metabolic Stress, Mitochondrial Injury, and Apoptotic Signaling

Cell death in neurodegenerative settings is often driven not by a single apoptotic trigger, but by the convergence of metabolic stress, mitochondrial dysfunction, redox imbalance, and inflammatory injury [185]. In this context, CoA and acetyl-CoA biology are relevant because mitochondrial bioenergetic failure can lower cellular stress tolerance and shift cells toward intrinsic death pathways. Experimental work outside the pantethine field shows that depletion of acetyl-CoA can precipitate severe oxidative and metabolic stress, culminating in loss of viability and apoptosis-like death [186]. Likewise, mitochondrial oxidative injury can promote membrane depolarization, cytochrome c release, and downstream caspase activation across diverse experimental systems [187,188]. Although these studies were not conducted in pantethine-treated neural models, they show that CoA-linked metabolic failure can converge on intrinsic apoptotic signaling [189,190,191,192].

In CoASY-related neurodegeneration models, mitochondrial damage, reduced ATP production, impaired respiratory-chain function, and apoptosis occur together, supporting energy failure and mitochondrial injury as upstream drivers of cell loss when CoA metabolism is compromised [124,193]. Pantethine is therefore relevant because it may reduce mitochondrial and redox conditions that favor cell death, not because it has been conclusively shown to block a defined apoptotic cascade in neurons across diseases [97].

#### 2.6.2. Evidence Linking Pantethine to Cell Survival Mechanisms

Direct evidence that pantethine itself modulates cell survival is limited [194]. The strongest support is indirect and comes from models in which pantethine or closely related CoA-restoring strategies reduce upstream drivers of cell death, including oxidative damage, mitochondrial dysfunction, and metabolic insufficiency [50]. In PKAN-related systems, pantethine rescue in Drosophila improves CoA levels, mitochondrial function, locomotor phenotype, and survival, indicating that correction of metabolic insufficiency can be accompanied by preservation of tissue integrity and reduced neurodegenerative progression [36,101].

Evidence from cysteamine and cystamine is relevant only as indirect mechanistic context because these aminothiols differ from pantethine in metabolism, pharmacokinetics, and tissue exposure [43]. Their reported effects on oxidative stress, neurotrophic signaling, apoptosis-related markers, and proteostasis therefore should not be attributed directly to pantethine [195]. Current pantethine-specific evidence more strongly supports an upstream contribution to cell survival through improved CoA-linked metabolism, mitochondrial function, and redox homeostasis [59]. Direct modulation of Bax, effector caspases, autophagic flux, or aggregate clearance by pantethine in disease-relevant neural systems remains unproven [196].

### 2.7. Glial–Neuronal Interactions and Metabolic Coupling

#### 2.7.1. Glial–Neuronal Crosstalk in Neurodegenerative Disease

Neurons function within a tightly coordinated cellular network in which astrocytes and microglia regulate metabolic support, synaptic homeostasis, and inflammatory tone [197,198,199]. Astrocytes contribute to substrate supply, neurotransmitter clearance, and local redox buffering, whereas microglia survey tissue integrity and respond to injury, aggregated proteins, and altered metabolites [198,199]. Under physiological conditions, these interactions support neuronal stability [199]. In neurodegenerative disease, however, glial–neuronal communication often shifts toward a maladaptive state in which inflammatory signaling, altered substrate handling, and mitochondrial stress reinforce one another rather than resolving injury [200]. During aging, these maladaptive glial states may additionally impair neurogenic niches and synaptic plasticity, linking chronic inflammation to reduced regenerative and cognitive resilience [116].

Astrocytes are central to glucose utilization, lactate provision, glutamate recycling, and antioxidant support, while microglia regulate cytokine networks and the metabolic state of neighboring cells [199]. Disturbances in mitochondrial function, iron handling, or CoA-dependent metabolism can therefore affect both neurons and the surrounding glial environment [201]. Direct pantethine-mediated reprogramming of glial–neuronal coupling in vivo has not yet been established.

#### 2.7.2. Potential Intersections Between Pantethine and Glial Metabolic Support

Pantethine-relevant evidence is strongest in astrocyte-centered AD models [37]. In 5xFAD astrocytes, pantethine improved abnormalities in glycolysis and TCA activity and reduced IL-1β [46]. These data show modification of astrocytic metabolic and inflammatory states under disease-like conditions but do not establish restoration of glial–neuronal coupling at the circuit level [123,132,202]. The same study reported increased hypoxia-inducible factor 1 alpha (HIF-1α) [46]. This may reflect an adaptive response linking metabolic stress, redox regulation, and astrocyte survival [203,204], but current evidence does not establish pantethine as a precise regulator of HIF-1α dynamics in the brain [205]. Pantethine may influence HIF-1α-linked metabolic remodeling under amyloid-related stress, but this remains provisional [104,206].

PKAN and related CoA-biosynthesis disorders provide a second, mechanistically stronger context for considering glial involvement [124]. Reviews of PKAN pathogenesis and treatment emphasize that CoA deficiency affects TCA activity, lipid metabolism, and mitochondrial function in ways that could plausibly alter both neuronal and astrocytic physiology [29,124,207]. These findings indicate that glial pathology is an active component of PKAN rather than a purely secondary consequence [28]. Astrocytes are metabolically abnormal, iron-loaded, and capable of worsening neuronal injury. Complementary evidence from COASY protein-associated neurodegeneration shows that patient-derived astrocytes exhibit greater iron accumulation and lipid peroxidation than matched fibroblasts, together with mitochondrial morphological abnormalities, impaired vesicular trafficking, ferroptosis-related changes, and cellular senescence, suggesting that astrocytes may reproduce the disease-relevant CoA-deficiency phenotype more faithfully than peripheral cells [97]. In that context, CoA supplementation reduces iron overload and cell death, supporting the broader idea that CoA restoration may have consequences for glial as well as neuronal survival [27,97].

Additional work reinforces this systems view [87]. A Pank2 mutant mouse displays region-specific CoA deficiency with abnormalities in iron handling, dopamine metabolism, and mitochondrial enzyme activity, all of which are corrected by oral 4′-phosphopantetheine [50]. Parallel studies show that multiple disorders, including PKAN and CoPAN, converge on a CoA, mtACP, and PDH axis that links impaired CoA homeostasis to reduced lipoylation and defective mitochondrial metabolism [28,51]. Neuronal Pank1 and Pank2 knockout mice also show detectable cerebral metabolic derangement by magnetic resonance spectroscopy, including altered glutamate, glutamine, lactate, and N-acetylaspartate ratios, findings that are reversible with a BBB-penetrant PANK activator [208]. Taken together, these studies support the idea that CoA deficiency disrupts the metabolic environment shared by neurons and glia, although they do not isolate pantethine-specific effects on astrocyte–neuron lactate exchange, glutamate transport, or microglial state transitions [198,199,209].

**Figure 4:**
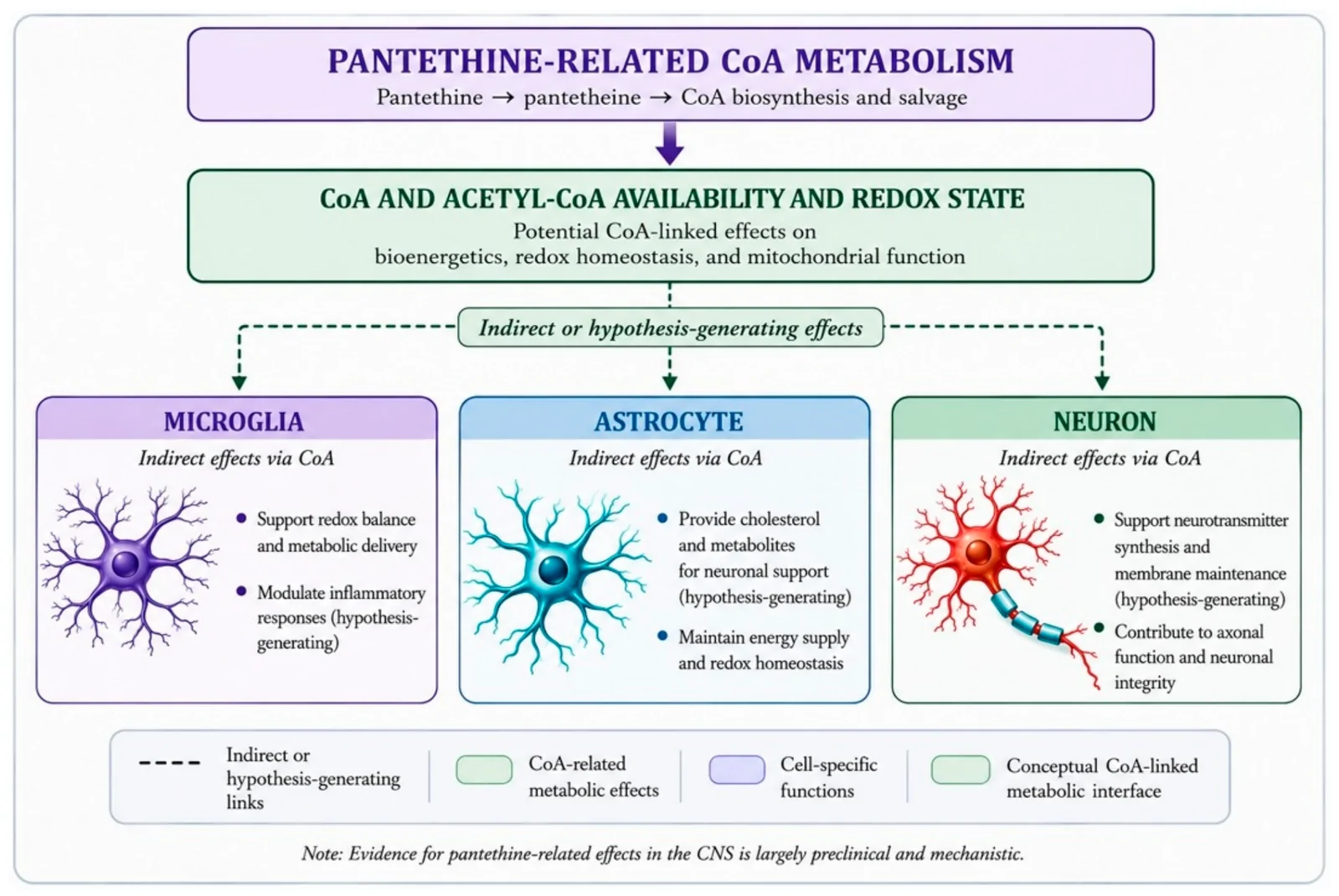
Proposed integrated actions of pantethine on glial and neuronal metabolism. Schematic representation of potential interactions of pantethine with microglial, astrocytic, and neuronal pathways. Pantethine may intersect with CoA- and GSH-dependent processes involved in redox regulation, energy metabolism, neurotransmitter and lipid synthesis, axonal integrity, and cellular homeostasis. These proposed effects are context-dependent and should be interpreted as hypothesis-generating rather than as established causal mechanisms.

Pantethine itself also shows rescue in Drosophila PKAN models, where it restores CoA, improves mitochondrial function, rescues brain structure, and improves locomotion and lifespan [36]. Combined supplement studies in PKAN fibroblasts and small patient cohorts report increased PANK2, mtACP, and PDH activity, together with clinical stabilization in selected cases [38]. These findings are compatible with improved metabolic support at the cellular level, but they remain indirect with respect to glial–neuronal coupling. They suggest that pantethine may help correct an upstream metabolic bottleneck that affects multiple cell types, yet they do not directly demonstrate restoration of astrocyte–neuron substrate exchange or microglia–astrocyte communication in vivo [36]. These putative links between glial metabolism, neuronal metabolism, and pantethine-related metabolic support are summarized schematically in Figure 4.

#### 2.7.3. Current Evidence Gaps and Hypothesis-Generating Perspectives

Evidence linking pantethine to glial–neuronal metabolic coupling remains largely inferential. Glial dysfunction is well documented in CoA-related neurodegeneration, particularly PKAN, where astrocytes show iron overload, respiratory defects, and reactive phenotypes [28,97]. Pantethine or related CoA-restoring strategies improve selected metabolic and inflammatory abnormalities in 5xFAD astrocytes and PKAN-related models [36,38,45,46,50,101], but direct restoration of lactate shuttling, glutamate clearance, or coordinated microglia–astrocyte phenotype transitions in vivo has not been demonstrated [123,131].

The available astrocyte data support HIF-1α as a possible adaptive node linking metabolic stress and inflammatory signaling [46], but do not establish precise pantethine-dependent regulation of HIF-1α in the brain. Direct evidence for pantethine-specific modulation of glial metabolic support remains limited and disease-context dependent [132,202].

Pantethine may affect glial–neuronal biology indirectly through CoA-linked metabolism, mitochondrial function, and inflammatory balance, particularly in PKAN-proximal contexts. Whether this restores cell–cell metabolic coupling in AD, PD, or other common neurodegenerative disorders remains uncertain. Coculture systems, cell-type-resolved metabolomics, and in vivo models can test these interactions directly.

### 2.8. Translational Relevance and Clinical Boundaries

#### 2.8.1. Why Pantethine Is Most Directly Relevant in PKAN

Translation should follow the evidence hierarchy established in Section 2.2. PKAN remains the most disease-proximal context, although pantethine is still investigational, whereas AD and PD should remain exploratory until CNS exposure, target engagement, and clinical efficacy are better established.

#### 2.8.2. Pharmacological, Biomarker, and Trial Design Challenges

Several obstacles currently limit translation. First, CNS exposure remains uncertain [91]. Oral pantethine is metabolically labile, and it is still unclear to what extent intact pantethine, pantetheine, cysteamine, or other downstream products mediate the observed biologic effects in brain tissue. Second, human trial data are limited. In the pediatric open-label PKAN study, pantethine was well tolerated and progression may have slowed, but motor scales and serum CoA did not significantly improve, underscoring the gap between preclinical rationale and clinical efficacy [56]. Preclinical development should also incorporate time-resolved behavioral safety testing, since related neuroactive compounds can show transient motor effects that are missed by single-time-point assessments [72,210]. Third, disease heterogeneity matters. In PD, related translational barriers include diagnostic imprecision, inconsistent biomarker standards, and poor alignment between mechanistic findings and clinically meaningful endpoints [108]. Patient-specific response may depend on mutation class, residual PANK2 activity, metabolic state, and degree of established neurodegeneration [97,100,211]. Experience from systems psychiatry similarly shows that acknowledged biological heterogeneity must be incorporated directly into biomarker selection, patient stratification, and trial design rather than treated as a post hoc explanation for variable response [183,212,213].

Translational studies therefore require biomarkers beyond clinical observation alone, prioritized according to mechanistic proximity to the CoA-pathway defect. In PKAN, the most proximal target-engagement measures include mtACP phosphopantetheinylation and the status of related mitochondrial phosphopantetheinyl proteins [53,54]. These should be followed by CoA and acyl-CoA pools, PDH lipoylation or activity, complex I function, and mitochondrial respiration as intermediate pathway readouts [53,54,92]. Broader metabolic and redox responses, including acylcarnitine profiles and glutathione redox status, can provide complementary evidence, whereas neurofilament light, inflammatory biomarkers, imaging, and cerebrospinal fluid measures primarily reflect downstream tissue injury or disease response [214,215,216]. Complementary metabolomic profiling may further characterize TCA-cycle and other metabolic changes accompanying target engagement, while longitudinal sampling can help capture time-dependent systemic variation [217,218,219]. This hierarchy would help distinguish direct pathway engagement from secondary biological effects [216,218,220]. Without such measures, negative or equivocal trials risk being uninterpretable, because lack of clinical response may reflect inadequate CNS delivery, wrong patient selection, insufficient dose, or failure to affect the intended pathway [24,97,100]. Multimodal biomarker integration and carefully calibrated machine-learning approaches may further improve patient stratification, provided that predictive models undergo external validation and demonstrate added clinical value beyond conventional assessment [221]. Together, these translational barriers define the research priorities considered in Section 3.

## 3. Critical Synthesis, Limitations, and Future Directions

### 3.1. What the Literature Supports Most Strongly

Taken together, the literature supports the strongest mechanistic coherence in inherited CoA-biosynthesis disorders, while translation remains constrained by unresolved active-species pharmacology, CNS exposure, and sparse human efficacy data. AD and PD therefore remain exploratory disease contexts rather than established therapeutic indications.

### 3.2. Major Limitations of the Current Evidence Base

The principal limitations arise from dependence on preclinical systems, sparse human efficacy data, unresolved active-species and CNS-exposure questions, and substantial methodological heterogeneity.

Another limitation is the continued overreliance on preclinical and cell-based systems. In vitro studies, fibroblast models, Drosophila systems, and mouse models have all contributed important mechanistic insight, yet they cannot fully reproduce the complexity of human neurodegeneration, especially in disorders such as AD and PD, where disease progression unfolds across multiple cell types, long time scales, and heterogeneous biological states. This is particularly relevant for redox biology, where Table 2 shows that the available evidence spans both brain-relevant and peripheral experimental systems, requiring caution when inferring direct neuroprotective effects from non-neural models. Even within PKAN, preclinical rescue does not automatically translate into robust clinical improvement [38,45].

Direct human efficacy data remain limited and methodologically constrained. The small open-label pediatric pantethine trial in PKAN supports tolerability and raises the possibility of slowed progression, but it did not show clear improvement in major motor scales or serum CoA [56]. This leaves several interpretations open, including inadequate CNS exposure, insufficient dosing, inappropriate biomarkers, or treatment at a stage when neuronal injury was already too advanced. At present, the human literature is too sparse to define pantethine as clinically effective, even in the disease context where its mechanistic rationale is strongest.

Another limitation concerns pharmacologic ambiguity. The active molecular species remains uncertain. Pantethine can be reduced or undergo disulfide exchange to form pantetheine, which is subsequently hydrolyzed by pantetheinases to pantothenate and cysteamine, and the degree to which each contributes to CNS-relevant effects is unresolved. Related to this, BBB penetration and actual neural target engagement remain insufficiently characterized. This limitation parallels a wider problem in neurotherapeutic development, where inconsistent exposure metrics, limited use of human-relevant BBB models, and insufficient comparison of delivery platforms can make it difficult to distinguish pharmacodynamic failure from inadequate brain delivery [90]. This is especially important because systemic metabolic effects should not be conflated with direct CNS rescue.

Additional limitations are methodological. Studies differ widely in outcome measures, dosing strategies, and mechanistic endpoints, limiting comparability across the literature. Some positive findings arise from combination regimens rather than pantethine monotherapy, making attribution difficult. As summarized in Table 2, oxidative and antioxidant findings are also distributed across heterogeneous model systems, which limits direct comparability and complicates interpretation of CNS-specific relevance. Disease heterogeneity also matters. Mutation class, residual enzyme activity, metabolic status, inflammatory state, age, and disease stage are all likely to influence responsiveness, yet these variables are rarely integrated systematically into study design. Finally, several areas of the literature, particularly glial–neuronal coupling and apoptosis-related mechanisms, remain vulnerable to overinterpretation, because mechanistic interpretations sometimes rest on indirect rather than direct pantethine-specific evidence. These limitations define the boundary between current evidence and future validation.

### 3.3. Future Directions for Mechanistic and Translational Research

Future research should prioritize disease-specific studies centered on demonstrable pathway engagement. In PKAN, genotype-aware designs should distinguish mutation class and residual PANK2 activity, while patient-derived fibroblasts, induced neurons, and neuron–astrocyte systems should be used to identify biologically plausible responders before clinical testing [27,100,211,212].

Pharmacokinetic and biomarker validation should proceed in parallel. Studies should quantify intact pantethine and relevant metabolites, distinguish peripheral from CNS exposure, and relate these measurements to proximal CoA-pathway target engagement [90,210]. In PKAN, biomarker assessment should begin with mtACP phosphopantetheinylation and related mitochondrial phosphopantetheinyl proteins, followed by CoA and acyl-CoA status, PDH and complex I function, and mitochondrial respiration. Broader metabolic, redox, inflammatory, imaging, electrophysiological, and clinical-response markers should then be interpreted as progressively more downstream measures of biological or disease response [50,51,160]. Multimodal models may assist responder stratification, but they require preregistration, external validation, and evidence of added clinical value [119,165,221]. Cell-type-resolved and longitudinal models are also needed to determine whether CoA-related interventions coordinate mitochondrial, redox, and inflammatory responses across neural and glial compartments [102].

For AD and PD, studies should remain explicitly exploratory and should compare pantethine with newer CoA-pathway strategies rather than assuming equivalence among interventions [108,207]. Adequately controlled comparative studies would be more informative than additional descriptive reports. Clinical development should therefore be restricted to settings in which the active molecular species, reached compartment, responder phenotype, and biomarker evidence of pathway engagement are prespecified [88,213].

## 4. Conclusions

Pantethine remains an investigational CoA-related metabolic candidate rather than an established neuroprotective therapy. Its mechanistic relevance lies at the intersection of CoA biology, mitochondrial bioenergetics, redox homeostasis, and inflammatory-metabolic stress. The strongest rationale for pantethine-related intervention is in PKAN and related inherited CoA-biosynthesis disorders, where pathway disruption is proximal to pathogenesis and CoA-restoring strategies have the clearest mechanistic support. Evidence in AD and PD remains indirect, predominantly preclinical, and hypothesis-generating, and does not establish disease modification. Major pharmacologic uncertainties also remain, including the active molecular species, CNS exposure, and biomarker-defined target engagement in humans. Clinical relevance therefore depends on rigorous, biomarker-guided, disease-specific validation.

## Figures and Tables

**Table 1:** Summary of pantethine-related evidence across mechanistic domains and disease contexts. Mechanistic/disease proximity is distinguished from experimental model characteristics and CNS relevance. The proximity categories describe the relationship of each finding to the disease mechanism and should not be interpreted as a formal grading of study quality.

Domain/Model	Principal Finding	Mechanistic/Disease Proximity	CNS/Human Relevance	Key Limitation	Ref.
Pharmacology and active species: pantethine metabolism in humans and ex vivo brain tissue	Rapid hydrolysis and context-dependent conversion indicate that parent pantethine may not be the dominant active species in vivo.	Mechanistically direct for pantethine pharmacology; not disease-specific.	Human metabolic data are available, but direct CNS exposure remains uncertain.	CNS exposure and the relative contributions of pantethine, pantetheine, and cysteamine remain unresolved.	[42,43,44]
PKAN and inherited CoA-biosynthesis disorders	CoA-pathway rescue is most coherent in models with primary CoA-pathway dysfunction, with improvements in iron handling, mitochondrial readouts, and survival-related phenotypes.	Disease-proximal.	Strong preclinical CNS and neuronal relevance; limited human therapeutic evidence.	Not all rescue studies isolate pantethine from related intermediates or combination regimens, and clinical translation remains limited.	[36,38,45]
Neuroinflammatory signaling: 5xFAD astrocytes and experimental autoimmune encephalomyelitis	Pantethine reduces selected inflammatory outputs, including IL-1β-related changes in astrocytes and TNF-α, GM-CSF, and IL-17A in encephalitogenic immune cells.	Disease-relevant.	Mammalian astrocyte and neuroinflammatory models provide CNS-relevant preclinical evidence; direct human CNS evidence is lacking.	Direct evidence for microglial reprogramming, IL-8 modulation, or broad CNS cytokine-network remodeling remains limited.	[34,46,47]
Oxidative stress and redox homeostasis: neurotoxicity and PKAN-related models	Pantethine or related CoA-linked precursors improve glutathione redox balance, reduce protein glutathionylation, and attenuate oxidative damage in selected models.	Disease-relevant.	Brain-relevant preclinical evidence is available, but human CNS efficacy has not been established.	Some supporting studies use panthenol rather than pantethine, and non-neural redox effects should not be equated with neuronal protection.	[48,49]
Mitochondrial dysfunction and bioenergetics: PKAN-linked models	CoA-related interventions improve CoA availability, respiration, membrane potential, PDH and complex I activity, and survival-related phenotypes in disease-proximal systems.	Disease-proximal.	Strong mechanistic and CNS-relevant preclinical evidence; direct clinical evidence remains limited.	Generalization to common neurodegenerative diseases is unproven, and some of the strongest evidence involves 4′-phosphopantetheine or other CoA-pathway strategies rather than pantethine itself.	[36,49,50,51]
Cell-survival pathways: PKAN and aminothiol-informed neuroprotection	Pantethine may reduce upstream drivers of cell death by improving metabolic and redox conditions, but direct pantethine-specific anti-apoptotic mechanisms remain insufficiently established.	Mechanistically indirect.	Evidence is largely preclinical and partly derived from related aminothiols.	Evidence for pantethine-specific effects on Bax, cytochrome c, or caspase pathways is largely inferred or derived from cysteamine/cystamine studies.	[36,52,53]
Glial–neuronal interactions and metabolic coupling: 5xFAD and PKAN-related systems	Pantethine may influence glial metabolic support and inflammatory tone, but direct restoration of lactate shuttling, glutamate clearance, or coordinated cell–cell metabolic coupling has not been demonstrated.	Hypothesis-generating.	CNS-relevant cellular systems support biological plausibility, but direct in vivo and human evidence is lacking.	Much of this domain rests on inferred links between CoA restoration and glial support functions.	[46,54,55]
Translational relevance: PKAN versus AD and PD	Translation is most strongly justified in PKAN; applications to AD and PD remain exploratory and should not be placed on equal evidentiary footing.	Disease-proximal for PKAN; disease-relevant/hypothesis-generating for AD and PD.	Limited human PKAN data are available; AD and PD evidence remains predominantly preclinical.	Human efficacy data are sparse, CNS target engagement is uncertain, and biomarker-defined trial design remains underdeveloped.	[26,38,56]

AD, Alzheimer’s disease; Bax, Bcl-2-associated X protein; CNS, central nervous system; CoA, coenzyme A; GM-CSF, granulocyte-macrophage colony-stimulating factor; IL-1β, interleukin-1 beta; IL-8, interleukin-8; IL-17A, interleukin-17A; iPSC, induced pluripotent stem cell; PANK2, pantothenate kinase 2; PD, Parkinson’s disease; PDH, pyruvate dehydrogenase; PKAN, pantothenate kinase-associated neurodegeneration; TNF-α, tumor necrosis factor alpha.

**Table 2:** Pantethine antioxidant and redox-modulating evidence in selected in vitro and in vivo models. Studies are grouped by model, concentration or dose, principal observation, CNS-specific relevance, and reference. Brain-relevant findings are distinguished from peripheral endothelial, fibroblast, erythrocyte, and irradiation models.

Model/Context	Intervention	Principal Redox Finding	CNS Relevance	Ref.
Human endothelial cells and NIH3T3 fibroblasts	Pantethine	Inhibited microparticle-associated oxidative and nitrosative stress.	Indirect. Demonstrates redox activity outside the nervous system but does not establish neuronal or glial protection.	[148]
Cancer-derived cellular models with differing p53/Bax status	D-pantethine	Cytoprotection was context dependent and was observed in wild-type HCT-116 cells but not in p53-null or Bax-null cells.	Indirect. Informative for redox-linked cellular survival pathways, not CNS-specific neuroprotection.	[151]
Murine systemic-sclerosis model	Pantethine	Reduced circulating endothelial microparticles and attenuated fibrosis, vascular abnormalities, and oxidative/endothelial stress.	Indirect. Supports systemic redox activity but has limited direct relevance to neurodegeneration.	[148]
Doxorubicin-associated oxidative injury in rats	Pantethine	Reduced erythrocyte oxidative stress but did not prevent the marked decrease in free CoA.	Indirect. Supports systemic redox modulation but not direct CNS rescue.	[151]
Irradiation-associated cataract model in rats	Pantethine	Produced only limited protection against cataract development compared with other antioxidant interventions.	Indirect. Ocular/systemic evidence; does not support neurodegeneration-specific efficacy.	[152]

ABCA1, ATP-binding cassette transporter A1; CNS, central nervous system; NIH3T3, mouse embryonic fibroblast cell line.

**Table 3:** Pantethine-associated mitochondrial and CoA-related effects in selected experimental and clinical systems. This table summarizes experimental and clinical evidence concerning pantethine and the closely related metabolite pantetheine in CoA-deficiency and neurodegeneration-relevant models. Direct mitochondrial findings are distinguished from CoA-related metabolic rescue and supportive clinical observations.

Model/Context	Intervention	Principal Mitochondrial/CoA Finding	Disease Relevance	Ref.
Patient-derived PKAN fibroblasts with residual PANK2 expression	Pantethine	Reduced iron accumulation, increased PANK2, mtACP, and NFS1 expression, and was associated with improvement in CoA-related mitochondrial markers in responsive cells.	Disease-proximal PKAN evidence, although obtained in peripheral patient-derived cells rather than CNS tissue.	[45]
Mouse brain mitochondria exposed to MPP^+^ ex vivo	Pantetheine, the reduced form of pantethine	Restored complex I activity under moderate MPP^+^ challenge and rescued mitochondrial respiration and ATP production under selected conditions; native pantethine was ineffective when added directly ex vivo.	Direct brain mitochondrial evidence, but toxin-based and ex vivo rather than a chronic neurodegenerative disease model.	[110]
dPANK/Fbl-depleted Drosophila Schneider S2 cells	D-pantethine	Restored cell growth and CoA levels; mitochondrial function was not the principal endpoint of this cellular experiment.	Disease-proximal genetic PANK-deficiency evidence, but non-mammalian and not a direct CNS preparation.	[36]
PPCS-deficient patient-derived fibroblasts, iPSCs, cardiac progenitors, and cardiomyocytes	Pantethine	Increased intracellular CoA and partially improved sarcomere organization, calcium handling, arrhythmic events, and contractile phenotypes.	Supportive inherited CoA-biosynthesis evidence, but non-neural and not evidence of CNS rescue.	[162,164]
Patients with genetically confirmed PPCS deficiency	Oral pantethine	Long-term treatment was associated with clinical and cardiac stabilization or improvement; direct longitudinal mitochondrial ultrastructural rescue was not demonstrated.	Human evidence in an inherited CoA-biosynthesis disorder, but predominantly cardiac rather than neuroprotective.	[162]
Pank2^−/−^ mice, including ketogenic-diet challenge	Pantethine	Increased mitochondrial oxygen consumption, preserved mitochondrial membrane potential in Pank2−/− neurons, and attenuated neuromuscular and mitochondrial pathology under metabolic challenge.	Direct mammalian PKAN-relevant brain and neuronal mitochondrial evidence.	[101]
MPTP/MPP^+^-related dopaminergic toxin model in mice	D-pantethine	Preserved brain mitochondrial complex I activity, increased brain glutathione, and supported ketone-related metabolism during dopaminergic mitochondrial injury.	Direct brain mitochondrial evidence in a PD-relevant toxin model, but not evidence from genetic or clinical PD.	[110]
Hypomorphic dPANK/fbl1 Drosophila	D-pantethine	Restored CoA levels, improved mitochondrial function and morphology, reduced neurodegeneration, improved locomotor performance, and extended lifespan.	Direct disease-proximal evidence in a genetic PKAN model, although non-mammalian.	[36]

Abbreviations: CoA, coenzyme A; CNS, central nervous system; NFS1, cysteine desulfurase; iPSC, induced pluripotent stem cell; PDH, pyruvate dehydrogenase; PKAN, pantothenate kinase-associated neurodegeneration; PPCS, phosphopantothenoylcysteine synthetase; PANK2, pantothenate kinase 2; mtACP, mitochondrial acyl carrier protein; MPP^+^, 1-methyl-4-phenylpyridinium; MPTP, 1-methyl-4-phenyl-1,2,3,6-tetrahydropyridine.

## Data Availability

No new data were created or analyzed in this study. Data sharing is not applicable to this article.

## Supplementary Materials

The following supporting information can be downloaded at: https://www.mdpi.com/article/10.3390/ijms27219926/s1.

## Abbreviations

The following abbreviations are used in this manuscript:

ABCA1	ATP-binding cassette transporter A1
AD	Alzheimer’s disease
AKT	protein kinase B
ATP	adenosine triphosphate
BBB	blood–brain barrier
CNS	central nervous system
CoA	coenzyme A
CoPAN	COASY protein-associated neurodegeneration
EAE	experimental autoimmune encephalomyelitis
ETC	electron transport chain
FADH_2_	reduced flavin adenine dinucleotide
GM-CSF	granulocyte-macrophage colony-stimulating factor
GSH	reduced glutathione
HIF-1α	hypoxia-inducible factor 1 alpha
IL-17A	interleukin 17A
IL-8	interleukin 8
iPSC	induced pluripotent stem cell
MPP^+^	1-methyl-4-phenylpyridinium
mPTP	mitochondrial permeability transition pore
MPTP	1-methyl-4-phenyl-1,2,3,6-tetrahydropyridine
mtACP	mitochondrial acyl carrier protein
NADH	reduced nicotinamide adenine dinucleotide
NADPH	reduced nicotinamide adenine dinucleotide phosphate
NBIA	neurodegeneration with brain iron accumulation
NF-κB	nuclear factor kappa B
NIH3T3	mouse embryonic fibroblast cell line NIH3T3
PANK	pantothenate kinase
PANK2	pantothenate kinase 2
PANK4	pantothenate kinase 4
PD	Parkinson’s disease
PDH	pyruvate dehydrogenase
PI3K	phosphoinositide 3-kinase
PKAN	pantothenate kinase-associated neurodegeneration
PPCS	phosphopantothenoylcysteine synthetase
ROS	reactive oxygen species
TCA	tricarboxylic acid
TNF-α	tumor necrosis factor alpha
VNN1	vanin 1
