# Supplementary material for: Pantethine and Neurodegeneration: A Coenzyme A-Centered Framework Linking Metabolism, Neuroinflammation, and Mitochondrial Dysfunction
Source: Int J Mol Sci. 2026 Nov 5;27(21):9926. doi: 10.3390/ijms27219926 (PMC13764653; doi:10.3390/ijms27219926)
Supplement: Supplementary file 1 [file ijms-27-09926-s001.zip › ijms-4585748-supplementary.pdf]

Table S1 retains the methodological detail available in the manuscript for the evidence clusters summarized in main-text Table 1. Where dose or exposure duration is not reported in the current summary table, it is not inferred here and should be completed from the cited primary studies if required by the journal.

Supplementary Table S1. Detailed methodological characteristics underlying the mechanistic evidence summary.

Domain/model	Experimental system	Intervention/compound	Dose/concentration	Route/exposure	Exposure duration	Study-specific methodological information	Principal finding	Ref.
Pharmacology and active species	Human dosing studies; ex vivo hippocampal/brain-tissue metabolism	Pantethine; pantetheine/cysteamine considered as downstream species	Varied across cited studies; not specified in the current summary table	Oral administration; ex vivo extracellular metabolism	Varied/not specified in the current summary table	Human and ex vivo studies evaluating pantethine hydrolysis, biotransformation, and brain-relevant extracellular metabolism.	Rapid hydrolysis and context-dependent conversion suggest that parent pantethine may not be the dominant active species in vivo.	[44–46]
PKAN and inherited CoA-biosynthesis disorders	Drosophila, mouse, fibroblasts, iPSC-derived neurons	Pantethine and related CoA-restoring strategies	Varied across studies	Dietary, oral, and cell supplementation	Varied across studies	Disease-proximal genetic and patient-derived systems with primary defects in CoA biosynthesis.	CoA-pathway rescue is most coherent in disease-proximal models, with improvements in iron handling, mitochondrial readouts, and survival-related phenotypes.	[38,40,47]

Neuroinflammatory signaling	5xFAD astrocyte cultures; murine and human encephalitogenic T-cell systems	Pantethine / CoA fueling	Varied across studies	In vitro and in vivo	Varied across studies	Astrocyte and autoimmune-neuroinflammation models used to examine inflammatory outputs under pantethine/CoA-fueling conditions.	Pantethine reduced selected inflammatory outputs, including IL-1β-related changes in astrocytes and TNF-α, GM-CSF, and IL-17A in encephalitogenic immune cells.	[36,48,49]
Oxidative stress and redox homeostasis	Rat brain regions; isolated brain mitochondria; PANK2-mutant cells; Drosophila	Pantethine or related CoA-linked precursors	Varied across studies	Oral, intragastric, and in vitro exposure	Varied across studies	Brain-relevant and PKAN-related systems examining glutathione balance, protein glutathionylation, and oxidative damage.	Selected CoA-linked interventions improved glutathione redox balance, reduced protein glutathionylation, and attenuated oxidative damage.	[50–52]
Mitochondrial dysfunction and bioenergetics	Drosophila; Pank2 models; fibroblasts; patient-derived cells	Pantethine and related CoA-restoring interventions	Varied across studies	Dietary, oral, and cell supplementation	Varied across studies	Disease-proximal systems with direct measurements of CoA-linked mitochondrial function.	Improved CoA availability, respiration, membrane potential, PDH/complex I activity, and survival-related phenotypes have been reported.	[38,53,54]

Cell-survival pathways	Drosophila and non-pantethine neural-injury models	Pantethine; cysteamine/cystamine-informed aminothiol interventions	Varied across studies	Dietary, oral, and experimental aminothiol exposure	Varied across studies	Evidence combines pantethine-related disease models with mechanistic literature on related aminothiols.	Pantethine may reduce upstream drivers of cell death through improved metabolic and redox conditions, but direct pantethine-specific anti-apoptotic mechanisms remain insufficiently established.	[38,55,56]
Glial-neuronal interactions and metabolic coupling	5xFAD astrocyte cultures; coculture systems; PKAN-derived cells	Pantethine-related supplementation	Varied across studies	In vitro; mixed supplementatio n contexts	Varied across studies	CNS-relevant cellular systems used to infer links between CoA restoration, astrocyte support, and inflammatory tone.	Pantethine may influence glial metabolic support and inflammatory tone, but direct restoration of lactate shuttling, glutamate clearance, or coordinated cell-cell coupling remains unproven.	[48,57,58]

Translational relevance: PKAN versus AD and PD	Open-label clinical trial; pilot combinations; preclinical comparisons	Oral pantethine; multimodal regimens	Varied across studies	Oral treatment and preclinical intervention paradigms	Varied across studies	Clinical and preclinical evidence considered separately according to disease context and target engagement.	Translation is most strongly justified in PKAN, whereas AD and PD remain exploratory and should not be presented on equal evidentiary footing.	[28,40,59]

Supplementary Table S2. Detailed methodological characteristics of redox studies summarized in Table 2.

Model/context	Intervention	Dose/concentration	Route/exposure	Duration	Study-specific methodological information	Principal redox finding	Ref.
Human pulmonary microvascular endothelial cells, human vascular endothelial cells, and NIH3T3 mouse fibroblasts	Pantethine	0–100 μM in endothelial cultures; 100 μM in NIH3T3 cells	In vitro cell-culture exposure	24 h in endothelial cultures; 4 h in NIH3T3 cells	Peripheral endothelial and fibroblast systems examining microparticle-associated oxidative and nitrosative stress.	Pantethine inhibited microparticle-associated oxidative and nitrosative stress.	[151]
HCT-116 isogenic lines (wild type, p53−/−, Bax−/−), MCF-7, Jurkat, HL-60, and HL-60/vinc cells	D-pantethine	1, 2.5, 25, and 50 μM	In vitro cell-culture exposure	Not specified in the current table	Cancer-derived cell models used to assess context dependence of cytoprotection and redox-linked viability.	D-pantethine protected wild-type HCT-116 cells but not p53-null or Bax-null cells, indicating context-dependent cytoprotection.	[154]
Six-week-old female BALB/c mice; systemic-sclerosis model	Pantethine	150 mg/kg/day	Oral	6 weeks	In vivo systemic-sclerosis model assessing microparticle shedding, vascular abnormalities, fibrosis, and oxidative/endothelial stress.	Reduced circulating endothelial microparticles and attenuated fibrosis, vascular abnormalities, and oxidative/endothelial stress.	[151]
Adult male Wistar CRL rats exposed to doxorubicin-related oxidative injury	Pantethine	400 mg/kg/day	Intragastric	5 days	In vivo hematologic oxidative-stress model assessing erythrocyte oxidative injury and free CoA.	Reduced oxidative stress in erythrocytes but did not prevent the marked fall in free CoA.	[154]
Male pigmented Long-Evans rats subjected to irradiation	Pantethine	1 g/kg, single dose	Intraperitoneal	Single dose 45 min before irradiation	In vivo irradiation model assessing cataract development relative to other antioxidant interventions.	Produced only a minimal delay in cataract development compared with other agents.	[155]

Supplementary Table S3. Detailed methodological characteristics of mitochondrial and CoA-related studies summarized in Table 3.

Model/context	Intervention	Dose/concentration	Route/exposure	Duration	Study-specific methodological information	Principal mitochondrial/CoA finding	Ref.
Patient-derived PKAN fibroblasts with residual PANK2 expression	Pantethine	5 μM; dose-response analysis at 1, 5, 10, 50, and 100 μM	Cell-culture exposure	20 days for the 5 μM protocol; dose-response duration not specified in the current table	Patient-derived peripheral cells with residual PANK2 expression; mitochondrial/CoA-related molecular markers were examined.	Reduced iron accumulation, increased PANK2, mtACP, and NFS1 expression, and was associated with improvement in CoA-related mitochondrial markers in responsive cells.	[47]
Isolated brain mitochondria from control mice exposed to MPP+	Pantetheine, the reduced form of pantethine	0.5 or 1 mM pantetheine under MPP+ challenge	Ex vivo mitochondrial incubation	Not specified in the current table	Isolated brain mitochondria exposed to graded MPP+ challenge; native pantethine was also tested directly ex vivo.	At 1 mM, pantetheine restored complex I activity under moderate MPP+ challenge and rescued respiration and ATP production under selected conditions; native pantethine was ineffective when added directly ex vivo.	[113]
Drosophila Schneider S2 cells with dPANK/Fbl depletion	D-pantethine	100 μM	Cell-culture exposure	Not specified in the current table	PANK-deficient Drosophila cell model; cell growth and CoA levels were principal endpoints.	Pantethine restored cell growth and CoA levels; mitochondrial function was not the principal endpoint.	[38]
PPCS-deficient patient fibroblasts, iPSCs, cardiac progenitor cells, and iPSC-derived cardiomyocytes	Pantethine	50, 150, and 500 μM for CoA measurements; 100 μM for 2D cardiomyocytes; 500 μM in 3D heart-patch experiments	Cell-culture exposure	5 days for the 100 μM 2D cardiomyocyte protocol; other durations not specified in the current table	Patient-derived cellular and cardiac models of inherited PPCS deficiency.	Increased intracellular CoA and partially improved sarcomere organization, calcium handling, arrhythmic events, and contractile phenotypes.	[164]

Patients with genetically confirmed PPCS deficiency	Oral pantethine	F1.II: 450 mg/day, subsequently 900 mg/day; F5:IV.1: 600 mg/day; F6:IV.1 and F6:IV.4: approximately 15 mg/kg/day	Oral	Long-term treatment; exact duration varied by patient and is not specified in the current table	Human PPCS-deficiency cases with predominantly cardiac manifestations.	Long-term pantethine treatment was associated with clinical and cardiac stabilization or improvement; direct longitudinal mitochondrial ultrastructural rescue was not demonstrated.	[164]
Pank2−/− mice and Pank2+/+ controls, including ketogenic-diet challenge	Pantethine	15 mg/kg/day	Drinking water	Not specified in the current table	Mammalian PKAN-relevant model with brain mitochondrial and neuronal measurements, including metabolic challenge.	Increased mitochondrial oxygen consumption, preserved mitochondrial membrane potential in Pank2−/− neurons, and attenuated neuromuscular and mitochondrial pathology under ketogenic challenge.	[104]
Male C57BL/6 mice exposed to 2′-methyl-MPTP	D-pantethine	15 mg/mouse/day	Intraperitoneal injection	Two 5-day treatment periods before and after toxin exposure	PD-relevant dopaminergic toxin model with brain mitochondrial, glutathione, and ketone-related metabolic endpoints.	Preserved brain mitochondrial complex I activity during MPP+ challenge, increased brain glutathione, and enhanced ketone-related metabolism.	[113]
Hypomorphic dPANK/fbl1 mutant Drosophila	D-pantethine	1.6 mg/mL in food	Dietary supplementation	Not specified in the current table	Genetic Drosophila model of PANK deficiency with CoA, mitochondrial, neurodegeneration, locomotor, and lifespan endpoints.	Restored CoA levels, improved mitochondrial function and morphology, reduced neurodegeneration, improved locomotor performance, and extended lifespan.	[38]
